# Synchronizing rock clocks in the late Cambrian

**DOI:** 10.1038/s41467-022-29651-4

**Published:** 2022-04-13

**Authors:** Zhengfu Zhao, Nicolas R. Thibault, Tais W. Dahl, Niels H. Schovsbo, Aske L. Sørensen, Christian M. Ø. Rasmussen, Arne T. Nielsen

**Affiliations:** 1 Department of Geosciences and Natural Resource Management, University of Copenhagen, DK-1350 Copenhagen, Denmark; 2 GLOBE institute, University of Copenhagen, DK-1350 Copenhagen, Denmark; 3 Geological Survey of Denmark and Greenland (GEUS), DK-1350 Copenhagen, Denmark

**Keywords:** Palaeoclimate, Stratigraphy, Geochemistry

## Abstract

The Cambrian is the most poorly dated period of the past 541 million years. This hampers analysis of profound environmental and biological changes that took place during this period. Astronomically forced climate cycles recognized in sediments and anchored to radioisotopic ages provide a powerful geochronometer that has fundamentally refined Mesozoic–Cenozoic time scales but not yet the Palaeozoic. Here we report a continuous astronomical signal detected as geochemical variations (1 mm resolution) in the late Cambrian Alum Shale Formation that is used to establish a 16-Myr-long astronomical time scale, anchored by radioisotopic dates. The resulting time scale is biostratigraphically well-constrained, allowing correlation of the late Cambrian global stage boundaries with the 405-kyr astrochronological framework. This enables a first assessment, in numerical time, of the evolution of major biotic and abiotic changes, including the end-Marjuman extinctions and the Steptoean Positive Carbon Isotope Excursion, that characterized the late Cambrian Earth.

## Abstract (web summary)

The Cambrian is the most poorly dated period of the past 541 million years. Here, the authors present a new astronomical time scale, allowing for a first assessment, in numerical time, of the evolution of major biotic and abiotic changes that characterized the late Cambrian Earth.

## Introduction

During the late Cambrian, profound changes in the Earth’s oceanic physio-chemical conditions took place along with shifts in atmospheric oxygen levels^1^. These changes coincided with biotic turnover, and conspicuous perturbations in the global carbon cycle and marine redox landscape^2–8^. Precise temporal constraints are fundamental for understanding their timing, duration and links to causal mechanisms. However, compared to other Phanerozoic intervals, the ages of Cambrian stratigraphic boundaries are poorly resolved^9^ as dated bentonite beds are rare in the Cambrian stratigraphic record^10^. The ages currently available for the Cambrian stage boundaries were estimated simply by assuming that successive biozones represent equal time intervals^9^. This is unlikely, however, as evolutionary turnover rates are not constant and as the uniformity in palaeontological practice for biozonal designation varies across clades and geographical occurrence^9,11^. As a further complication, global biostratigraphic correlation is hampered by the pronounced faunal provincialism at this time^9^ and as a result, stage boundaries within the Cambrian system have proven particularly difficult to ratify by the International Union of Geological Sciences.

Cyclostratigraphy is a powerful tool for refining the geological time scale, but thus far applications in the early Palaeozoic have been relatively few^12,13^. Astronomically forced climate cycles expressed in sediments^14^, when tuned to an astronomical solution, yield a high-resolution astronomical time scale^15^. During the past two decades, the construction of an astronomical time scale has been well underway for the Cenozoic–Mesozoic eras^12,13,16^, assisted by orbital solutions to the solar insolation on Earth^17,18^. Despite the lack of complete orbital solutions prior to 50 Ma, the 405-kyr long orbital eccentricity, caused by gravitational interactions between Jupiter and Venus, is considered stable over most of Earth’s history, enabling it to be used as a reliable astronomical metronome^15–19^. Well-expressed orbital cycles have been found in 1.4 Ga Proterozoic marine sediments^20,21^, and in 2.48 Ga old banded iron formations of South Africa^22^. Apart from one recent study detailed below, previous studies of Cambrian cyclostratigraphy have mostly focused on whether orbital forcing can be recognized at all, and where present, only short intervals spanning a few 405-kyr cycles have been observed at widely disparate localities^23–26^.

**Fig. 1:**
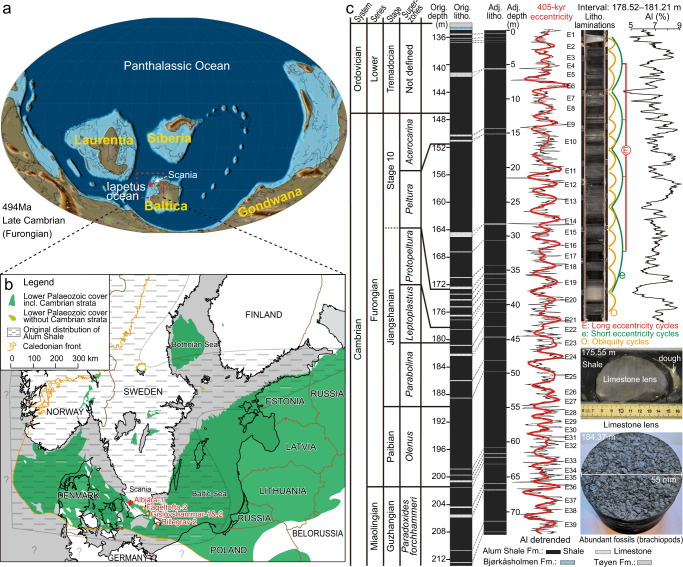
Summary of relevant geographical and stratigraphic data. **a** Late Cambrian (494 Ma) global palaeogeography^85^. **b** Location map showing the original distribution of the Alum Shale Formation in southern Scandinavia and the location of wells referred to (modified from ref. ^30^). **c** Biozonation, lithology and cyclostratigraphy of the Furongian interval including strata below and above in the Albjära-1 core, as well as representative core photos of the shale, a limestone lens and a bedding surface covered by fossils. The base of Stage 10 remains to be formally defined (see text for remarks), only the base defined by the FAD of *L. americanus* is plotted here. The 405-kyr eccentricity cycles in four subsets 0–18, 18–41, 41–55 and 55–74 m were extracted using Taner filters with centres (cut-offs) of 1.9 (1.3–3.3) cycles/m, 2.4 (1.5–4.2) cycles/m, 2.3 (1.0–5.0) cycles/m, and 1.8 (1.1–3.6) cycles/m, respectively. Adjusted depth was obtained by measuring from the Alum Shale Formation top downward after reducing the original limestone thickness to 20%. Lamination cycles are closely tracked by Al concentration variations (the 178.52–181.21 m interval is shown as an example).

Recently, Milankovitch-driven Cambrian cycles were recognized in two drill cores in southern Scandinavia, enabling the establishment of a floating astronomical time scale for a ~8.7 Myr interval across the Miaolingian–Furongian boundary^27^. Still, the lack of numerical age constraints has prevented independent time control and the establishment of a continuous, robust Cambrian temporal framework. Here, we describe astronomically forced cycles recorded by the aluminium content (1 mm resolution) in the upper part of the Alum Shale Formation, southern Sweden, calibrated to previous records from the same interval^27^. This new time framework is constrained by radioisotopic ages published for the late Cambrian–Early Ordovician^11,28,29^, and covers a stratigraphic interval from the Guzhangian (~499.9 Ma) to the early Tremadocian (~483.9 Ma) (Fig. 1c). Regional and global correlations are based on integrated constraints from biostratigraphy, gamma-ray log stratigraphy, carbon isotopes, as well as elemental chemostratigraphy.

## The Alum Shale Formation

The Baltoscandian platform was exceptionally flat and tectonically quiescent during the Cambrian, and active subsidence of this ancient craton was minimal^30^. The Alum Shale Formation was deposited from the Miaolingian (middle Cambrian) to the Early Ordovician (Tremadocian) in the offshore deeper parts of an extensive epicontinental sea. The facies originally blanketed all of western Baltica and roughly covered an area of about 1 × 10^6^ km^2^, from Finnmark in northernmost Norway to northern Poland in the south and from the Norwegian/Swedish Caledonides in the west to Estonia and the St. Petersburg area of Russia in the east (Fig. 1b). The formation thickens from ~20–25 m across much of Sweden to ~80–100 m in Scania (southern Sweden) and the Oslo region (southern Norway). The present study of the upper ~77 m of the Alum Shale Formation in the Albjära-1 drill core from Scania spans the Guzhangian to Tremadocian interval, and mainly comprises finely laminated, non-bioturbated organic-rich black shales deposited on the outer shelf (Fig. 1c). In this setting, deposition was continuous but varied with sea-level changes, being expanded in lowstand intervals and condensed in highstand intervals^31^. A detailed biostratigraphy, comprising nine Miaolingian–Furongian superzones subdivided into 31 zones based on trilobites and agnostoids and three Tremadocian zones based on graptolites, provides an important stratigraphic framework for comparison of sections across Scandinavia^31,32^. A detailed stratigraphic framework for the Albjära-1 drill core has been established (Supplementary Fig. 1) based on the correlation of fossils, gamma-ray log patterns, and δ^13^C_org_ chemostratigraphy from multiple localities in Scania (Sweden) and on Bornholm (Denmark)^8,33^.

## Aluminium as a palaeoclimate proxy

X-Ray fluorescence core scanning of the Albjära-1 core at 1 mm resolution yielded geochemical profiles for an array of chemical elements. Among them, we selected aluminium (Al) for cyclostratigraphic analysis because (i) Al is hosted mainly in aluminosilicates that are the predominant component of clay, and thus, highly affected by continental weathering processes^34^, (ii) Al is hosted in insoluble phases and, thus, less sensitive to diagenetic alteration^34,35^, and (iii) a previous study of other Alum Shale cores has found cyclic patterns in clay-bound elements^27^. Therefore, cyclic variations in the Al content of the Alum Shale are presumed useful as an indicator for palaeoclimatic changes. In general, warm and humid climates are associated with enhanced chemical weathering, precipitation and runoff, and thus intensified clay supply to the sea, causing higher Al concentrations in the marine sediments, and vice versa in colder and/or drier climates. In addition, the cyclic effect of weathering-induced Al concentrations was likely further amplified by pyrite dilution, e.g., via aeolian dust supply of iron into the ocean, which was enhanced during increased aridity and colder climates^27^.

## Results

The multitaper method (MTM) spectrum of the uncalibrated Al series through the entire stratigraphic interval shows dominant wavelengths of 1.79–2.90, 0.48–0.76, 0.13–0.22 and 0.08–0.11 m (Supplementary Fig. 4a), with ratios that fit well with those of the theoretical late Cambrian orbital parameters^36^ (see Supplementary Note 2 for details). Given the variable sedimentation rates revealed by our evolutive spectrogram (evolutive Fast Fourier transform [evoFFT]; Supplementary Fig. 4b), we conducted the cyclostratigraphic analysis in four subsets, viz. core intervals 0–18, 18–41, 41–55 and 55–74 m. These four intervals display robust peaks at ~1.85, ~2.44, ~2.78 and ~1.84 m, respectively, which are all interpreted as reflecting 405-kyr eccentricity cycles (Supplementary Fig. 4c), based on an average (compacted) sedimentation rate estimation of 4–5 mm/kyr in Scania (ref. ^37^; Supplementary Note 3). When 405 kyr is used to time-calibrate these stratigraphic cycles, the significant spectral peaks in addition to the 405-kyr peak indicate cycles with periods consistent with the theoretical ratio of the astronomical parameters for the late Cambrian (Supplementary Note 2). Variations in the sedimentation rate at 405-kyr scale also match well with the sedimentation rate map derived iteratively using the evolutionary correlation coefficient (eCOCO) algorithm (Supplementary Fig. 5) and variations observed in other parts of the Alum Shale basin^27^. The sedimentation rates are inversely correlated with late Cambrian sea-level reconstructions in the basin as should be expected^31^ (Supplementary Fig. 5). The MTM spectrum of the entire 405-kyr-calibrated time series displays significant spectral peaks at ~2.6 Myr, ~1.8 Myr, ~1.3 Myr, 405 kyr, ~108 kyr, ~30.9 kyr and ~17.1–20.9 kyr above the 99.9% confidence level (Supplementary Fig. 6a, b). These peaks are consistent with the major periods expected from the orbital forcing of solar insolation on the late Cambrian Earth^12,13,36^. Application of the Al-based age model to other lithogenic elements, silicon and titanium, further support this interpretation (Supplementary Fig. 6d–i). The obliquity component is significant (Supplementary Fig. 6b, e, h), which fits expectations since obliquity forcing of insolation increases polewards with pronounced expression at 60°–80° latitude^13^, and Baltica was located at ~60° S palaeolatitude at this time^38^. Amplitude modulations of the obliquity cycles reveal persistent periods at ~1.3 Myr (Supplementary Fig. 7), near the secular frequency (*s*_4_–*s*_3_) originating from Mars and Earth’s orbital inclination variations at ~1.2 Myr. This modulation is also an important feature in Cenozoic–Mesozoic sedimentary records^39^. This modulation periodicity has not been reported from the Cambrian before, but due to the extended duration of the interval studied here (~16 Myr) at a very high resolution (2–3 kyr), it could be detected and demonstrated as a significant component in our dataset. The collective array of cyclostratigraphic results, thus, confirms a strong insolation-forced imprint on the late Cambrian climate and on sedimentation in the Alum shale basin. More details on the cyclostratigraphic interpretation are provided in Supplementary Note 3.

## Discussion

### Comparison with Milankovitch cycles published for the late Cambrian

**Fig. 2:**
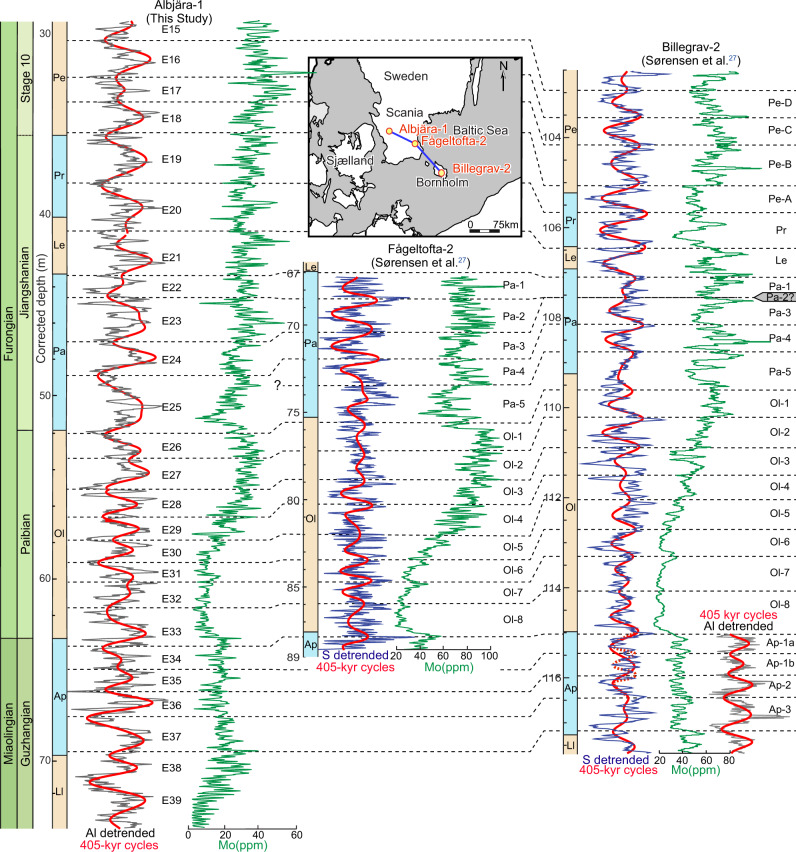
Stratigraphic correlation between the Albjära-1, Fågeltofta-2 and Billegrav-2 cores based on detrended Al and S signals, 405-kyr filtered outputs and molybdenum profiles. A 12-point (corresponding to 12 mm) smoothed Al series was used in the cyclostratigraphic analysis, but the Al and S series of the Albjära-1 core in this figure is smoothed with a window of 150 mm to visually emphasize the cycles. Mo curves from the three cores have the same smoothing window of 60 mm to visually correlate every 405-kyr cycle. All results shown for the Fågeltofta-2 and Billegrav-2 cores are from ref. ^27^ except for the additional Al cycle column in the *Agnostus pisiformis* Zone of the Billegrav-2 core. The Ap-1a and Ap-1b cycles refer to one Ap-1 cycle in ref. ^27^. The boundary between the *Agnostus pisiformis* and *Lejopyge laevigata* zones in the Billegrav-2 core was adjusted to ~117.3 m based on Mo correlations. The base of Stage 10 defined as in Fig. 1. Superzone/Zone abbreviations: Pe *Peltura*, Pr *Protopeltura*, Le *Leptoplastus*, Pa *Parabolina*, Ol *Olenus*, Ap *Agnostus pisiformis*, Ll *Lejopyge laevigata*.

Sørensen et al.^27^ recently reported Milankovitch cycles from a ~8.7 Myr interval across the Miaolingian–Furongian boundary in two Alum Shale drill cores, Fågeltofta-2 from eastern Scania (southern Sweden), and Billegrav-2 from Bornholm (Denmark). The 405-kyr cycles identified in the Albjära-1 core show overall an excellent match with these results. Although the previous cyclostratigraphic analysis focused on sulfur (S), a strong anti-correlation between the detrended Al and S signals (ref. ^27^, their Supplementary Fig. 5) facilitates correlation with the present study. Figure 2 shows a nearly perfect correlation between the three cores, which are constrained by biostratigraphy and molybdenum (Mo) trends in the overlapping intervals. The correlation points match twenty-two 405-kyr cycles (E16–E37) but with two discrepancies: in the lower *Parabolina* Superzone where one 405-kyr cycle (E25) in the Albjära-1 core correlates with two cycles (Pa-4 and Pa-5) in the Fågeltofta-2 and Billegrav-2 cores, and in the *Agnostus pisiformis* Zone, where two 405-kyr cycles (E34 and E35) in the Albjära-1 core correspond to only one cycle (Ap-1) in the Billegrav-2 core.

To investigate these discrepancies further, more detailed cyclostratigraphic analyses of the uncalibrated Al series from the lower *Parabolina* Superzone and the *A. pisiformis* Zone in the Albjära-1 core were conducted (Supplementary Fig. 8). The detailed analysis of the E25 interval of the Albjära-1 core confirms our initial interpretation (Supplementary Fig. 8a, b), but the possibility that one 405-kyr cycle is condensed or missing cannot be excluded, considering the sea-level lowstand conditions that prevailed during the deposition of the lower *Parabolina* Superzone^31^ (Supplementary Fig. 5), and the fact that the Pa-4 and Pa-5 cycles are well expressed in the corresponding interval of the Fågeltofta-2 and Billegrav-2 cores^27^ (Fig. 2). Although the 16-Myr record represented by the Albjära-1 section provides the most complete astrochronological record, a (possible) local hiatus in the lower *Parabolina* Superzone suggest that dates for boundaries below the *Parabolina* Superzone may carry an additional error, which is included in the calculated uncertainty of the ages of biozone and stage boundaries (see section “Establishing and testing a radioisotopically anchored late Cambrian astronomical time scale”). The new data show two well-expressed E34 and E35 cycles associated with higher-frequency cycles in the *A. pisiformis* zone (Supplementary Fig. 8c, d), matching two Al-based 405-kyr cycles in the Billegrav-2 core (labelled Ap-1a and -1b in Fig. 2), which are not recorded in the sulfur data from that core^27^. Apart from these two discrepancies, the antiphase relationship between the Al- and S-derived 405-kyr cycles is interrupted at cycle E37 (Ap-3 in the Billegrav-2 core) (Fig. 2). Detailed analysis of this interval revealed well-expressed higher-frequency oscillations (~32.7-kyr and ~18.5-kyr cycles) within the E37 cycle (Supplementary Fig. 8e, f), and this cycle can also confidently be correlated with that of the Al-based Ap-3 cycle in the Billegrav-2 core, as supported by correlation of the molybdenum profiles (Fig. 2). Therefore, the insolation forcing on Al may be more robust than S in this interval, because in the anoxic Alum Shale basin, S (hosted mainly in pyrite, FeS_2_) is affected by the iron supply to the basin via multiple plausible sources such as benthic iron shuttle, aeolian dust and clastic input^27,40^.

### Determination of the Cambrian–Ordovician boundary

**Fig. 3:**
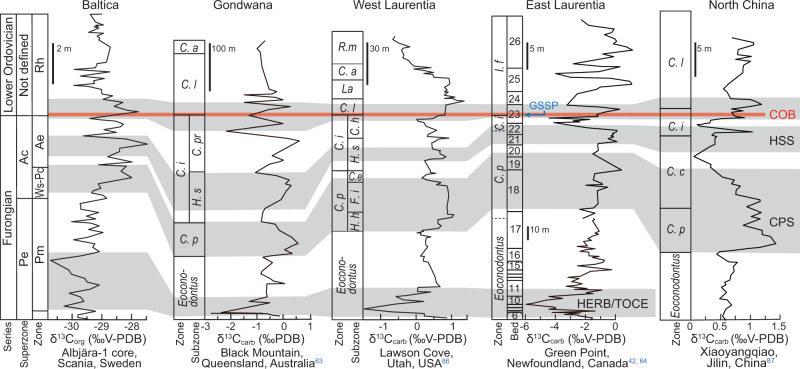
Global biozone and carbon isotope correlations across the Cambrian–Ordovician boundary. Carbon isotope excursions include the Hellnmaria-Red Tops Boundary Event/the Top of Cambrian Excursion (HERB/TOCE; refs. ^8,52,53,63–67,86^), *Hirsutodontus simplex* spike (HSS; refs. ^8,63,87^), *Cordylodus proavus* spike (CPS; refs. ^8,63,87^) and excursion at the Cambrian–Ordovician boundary (COB; refs. ^8,41,42^). Based on these excursions, the Cambrian–Ordovician boundary can be confidently determined for the Albjära-1 well, which is in accordance with the biostratigraphic data at hand. Ac *Acerocarina*, Pe *Peltura*, Rh *Rhabdinopora* spp., Ae *Acerocare ecorne*, Ws–Pc *Westergaardia scanica*, *Acerocarina granulata* and *Peltura costata*, Pm *Parabolina heres megalops*; *I. f* *Iapetognathus fluctivagus*, *R. m* *Rossodus manitouensis*, *C. a* *Cordylodus angulatus*, *La* *lapetognathus*, *C. l* *Cordylodus lindstromi*, *C. i* *Cordylodus intermedius*, *C. p* *Cordylodus proavus*, *C. pr* *Cordylodus prolindstromi, C. h* *Clavohamulus hintzei*, *H. s* *Hirsutodontus simplex*, *C. e* *Clavohamulus elongatus, F. i* *Fryxellodontus inornatus*, *H. h* *Hirsutodontus hirsutus*.

The Global Boundary Stratotype Section and Point for the Cambrian–Ordovician boundary (COB) has been ratified at Green Point, western Newfoundland, coinciding with the First Appearance Datum (FAD) of the conodont *Iapetognathus fluctivagus*, which is located just 4.8 m below the earliest occurrence of planktic graptolites^41^. This boundary coincides with a positive carbon isotope (δ^13^C_carb_) excursion, featuring a “double switch-back” at about the COB before reaching the maximum value of ~–2‰ in the lowermost Ordovician^41,42^. Hence, the COB is located at the rising limb of this positive excursion. Based on detailed carbon isotope correlations with records from Australia (Queensland), USA (Utah), Canada (Green Point section) and China (Jilin) (Fig. 3), the COB in the Albjära-1 core is inferred located at 146.86 m in the middle of this rising limb (corresponding to an adjusted depth of 11.18 m below the top of the Alum Shale Formation). This position is corroborated by a perfect correlation of the gamma-ray log and δ^13^C_org_ curve to the Gislövshammar-1 and Gislövshammar-2 wells in SE Scania (ref. ^8^; Supplementary Fig. 1). The biozonation has been investigated in great detail in the Gislövshammar-1 well^43,44^. The Albjära-1 core has been kept as intact as possible without splitting the core systematically for fossil investigations, and the first reliable record of the planktic graptolite *Rhabdinopora* is on an incidentally exposed core surface at 142.0 m. A few possible graptolite stipe fragments have, however, been observed at 146.63 m just above the COB (Supplementary Fig. 1). In the Gislövhammer cores, the first record of planktic graptolites is ~0.67 m above the COB as predicted by the δ^13^C_org_ isotope pattern (Supplementary Fig. 1).

### Establishing and testing a radioisotopically anchored late Cambrian astronomical time scale

**Fig. 4:**
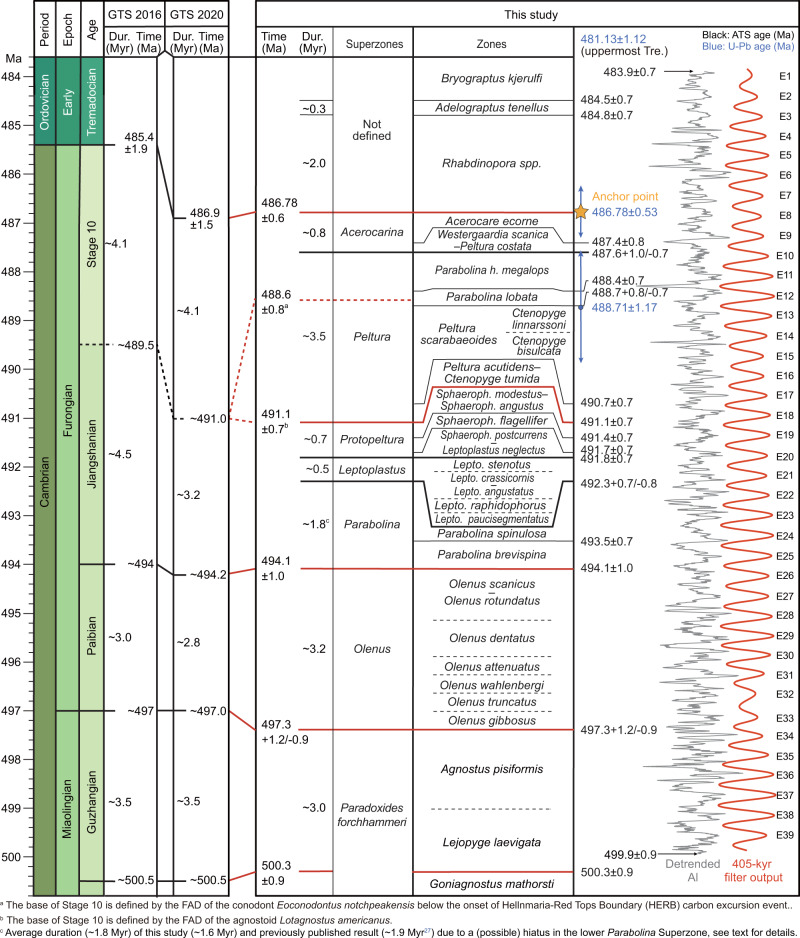
Comparison between the radioisotopically anchored astronomical time scale and the international chronostratigraphic time scales. The age model was anchored to the U-Pb age of 486.78 ± 0.53 Ma for the Cambrian–Ordovician boundary^11,47^, and supported by the 481.13 ± 1.12 Ma for the uppermost Tremadocian^28,47^, and 488.71 ± 1.17 Ma for the upper *Peltura scarabaeoides* Zone^29,47^. The 405-kyr long orbital eccentricity cycles (red curve) were extracted using a bandpass Taner filter (passband: 0.00247 ± 0.00055 cycles/kyr).

The COB age of 485.4 ± 1.9 Ma, published in the GTS2012^10^ and GTS2016^45^, was calculated by a spline fit of 26 radioisotopic age determinations through the late Cambrian–Early Devonian interval. In GTS2020, the number of radioisotopic dates increased to 49, and an age of 486.9 ± 1.5 Ma was recalculated for the COB^46^. Nonetheless, the general scarcity of stratigraphically well-constrained age determinations in the Cambrian creates a large uncertainty for the calculated COB age^10^. A U-Pb date of an ash bed from the uppermost Furongian *Acerocare ecorne* Zone at Bryn-llin-fawr, North Wales, located just ~4 m below the first occurrence of the graptolite *Rhabdinopora* (traditional index fossil for the base of the Ordovician), provides a precise numerical age constraint of 489 ± 0.6 Ma^11^. In GTS2020, this age was recalculated at 486.78 ± 0.53 Ma using a corrected U decay constant^47^. Anchoring our 405-kyr calibrated time series to this U-Pb date, which is well within the range of the calculated age (486.9 ± 1.5 Ma) based on a spline fit of 49 radioisotopic dates from the lower Palaeozoic (cf. GTS2020^46^), enables the construction of an anchored astronomical time scale for the late part of the Cambrian and the earliest Ordovician, spanning from 499.9 ± 0.9 to 483.9 ± 0.7 Ma (Fig. 4).

The astronomical time scale carries the following uncertainties: (i) the error of the 486.78 ± 0.53 Ma U-Pb dating of the COB; (ii) the uncertainty in precisely determining the position of the COB in the studied core based on δ^13^C_org_ data, as the rising limb of the COB positive excursion straddles a 0.29-m-thick interval (146.71–147.00 m), which corresponds to 0.05 Myr (i.e., ±0.03 Myr error if the COB is assumed located at 146.86 m in the middle of that interval); (iii) the assumption of a constant sedimentation rate between every two 405-kyr cycle minima; (iv) the uncertainty of spectral peak assignments in the cyclostratigraphic signal due to nonlinear climatic response that potentially caused variable time lags between orbital forcing and sedimentation cyclic expression; here, we follow the previously proposed assumption of ±0.10 Myr^27^; (v) the uncertainties in identifying the exact location of biozone or stage boundaries in the Albjära-1 core; these uncertainties in metres are translated into durations using the 405-kyr-derived sedimentation rate, and the resulting errors of which (labelled as e_bio_) range between 0.07 and 0.35 Myr, see Supplementary Note 1 for details; (vi) due to the single discrepancy between the analysis of the Fågeltofta-2 core (indicating 5 cycles in the *Parabolina* Superzone^27^) and that of the present study (4 cycles), we have adopted the average of the two studies (i.e., 4.5 cycles), and therefore added an additional error of ±0.20 Myr (half 405-kyr cycle) to all ages below the *Parabolina* Superzone. All in all, the uncertainties of dating the biozone and stage boundaries above and below the *Parabolina* Superzone are estimated to be 0.66+e_bio_ (=0.53 + 0.03 + 0.10+e_bio_) Myr and 0.86+e_bio_ (=0.53 + 0.03 + 0.10 + 0.20+e_bio_) Myr, respectively.

The radioisotopically anchored astronomical time scale (Fig. 4) is further constrained by two additional isotope dates. The first one is an adjusted U-Pb zircon date at 488.71 ± 1.17 Ma, based on a volcanic ash from Ogof-ddu, Criccieth, N. Wales^29,47^. This ash bed was located just 0.6 m above the first occurrence of *Ctenopyge linnarssoni* in the lower part of the *Peltura scarabaeoides* Zone^29^ according to Henningsmoen’s scheme^48^, but recently, the revised definition of the *P. scarabaeoides* Zone places it in the upper part of this zone^31^. The U-Pb age corroborates our calculated age range for the upper half of the *P. scarabaeoides* Zone (~488.7–489.7 Ma). The second adjusted U-Pb zircon age of 481.13 ± 1.12 Ma was reported from an uppermost Tremadocian K-bentonite in the Chesley Drive Group, McLeod Brook, at Cape Breton, Canada^28,47^. In Scania, the Alum Shale Formation is overlain by the Bjørkåsholmen Formation and the Tøyen Formation (Fig. 1c). The upper boundary of the Tremadocian Stage corresponds to a level somewhere in the lower part of Tøyen Formation, a few metres above the top of Alum Shale Formation. This is consistent with the estimated age of 483.9 ± 0.7 Ma for the top of the Alum Shale Formation, which is ~2.8 Myr older than the dated uppermost Tremadocian bentonite from Cape Breton. Hence, the few existing radioisotopic dates published for the studied interval fit with the time scale constructed on the basis of astronomical cycles (Fig. 4), corroborating the reliability of the age model and the feasibility of building an astronomical time scale for the late Cambrian.

**Fig. 5:**
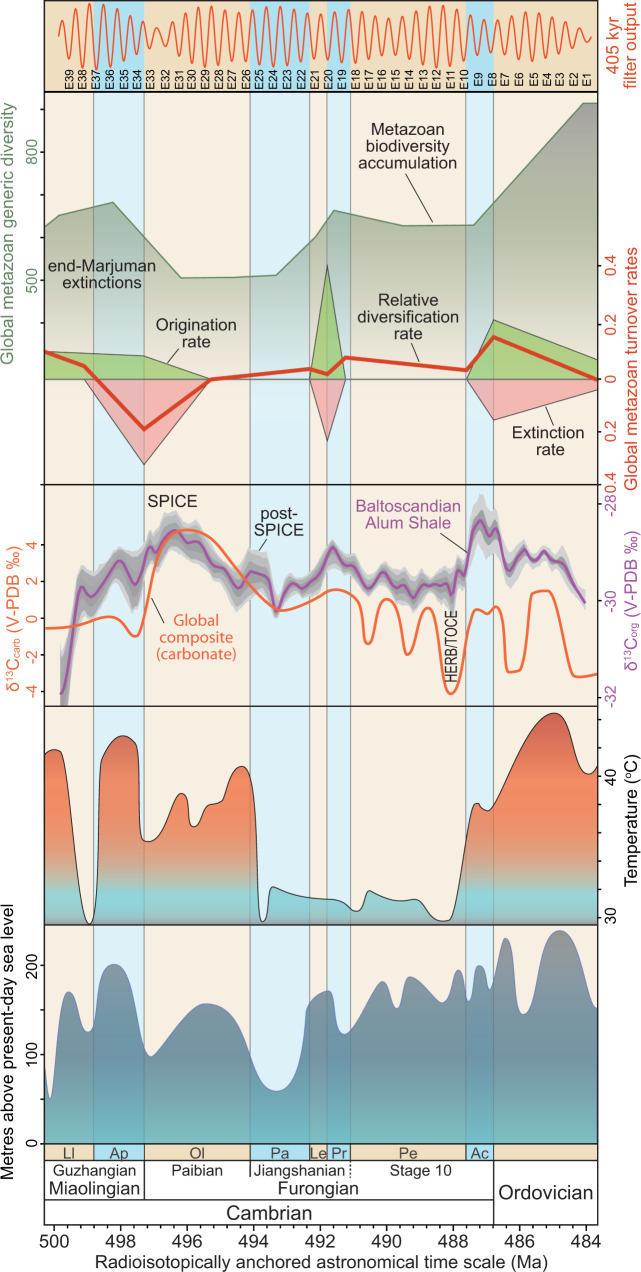
Temporal correlation of late Cambrian–earliest Ordovician biotic and abiotic events calibrated to the Baltoscandian radioisotopically anchored astrochronological framework. Elevated extinction rates coincide with extreme heat and high sea level, whereas the rapid Jiangshanian rebound occurs at ~492 Ma while temperatures were lower. Generic richness data based on refs. ^5,88^; δ^13^C_org_ data from the Albjära-1 core in this study and δ^13^C_Carb_ curve from GTS2020^49^. Temperature data based on ref. ^74^ with blue shading denoting the current tropical sea surface temperature range, red above that window. Sea-level curve adopted from ref. ^5^. The base of Stage 10 defined as in Fig. 1. Blue and orange shaded intervals represent Scandinavian biozones as shown in Fig. 2 except the abbreviation Ac *Acerocarina* Superzone.

The ages of the bases of the late Cambrian Jiangshanian, Paibian and Guzhangian stages are, respectively, calculated at 494.1 ± 1.0, 497.3 + 1.2/–0.9 and 500.3 ± 0.9 Ma. These dates agree well with the GTS2020 approximations of ~494.2, ~497.0 and ~500.5 Ma^49^. The base of Cambrian Stage 10 remains to be defined. Two levels are discussed as potential candidates, viz. the FAD of the agnostoid *Lotagnostus americanus*^50,51^ or the FAD of the conodont *Eoconodontus notchpeakensis* below the onset of Hellnmaria-Red Tops Boundary (HERB) carbon excursion event^52,53^. The lower boundary age of Stage 10, based on the FAD of the *L. americanus*, was estimated at ~489.5 Ma in the GTS2016^45^ and at ~491.0 Ma in the GTS2020^49^. This was done by assigning the recalculated 488.71 ± 1.17 Ma zircon date^47^ (erroneously stated to be redated as 490.1 ± 0.6 Ma in GTS2020^9^, see Supplementary Note 1 for details) to the base of the *Ctenopyge bisulcata* Subzone [now abandoned lower part of the *P. scarabaeoides* Zone^31^] and assuming a 1 Myr duration of the *L. americanus* Zone^9,54^. However, as remarked above, the dated ash derives from the upper part of revised *P. scarabaeoides* Zone^31^ and a 1 Myr duration of the *L. americanus* Zone is also a highly uncertain approximation. The lowest record of *L. trisectus* [= *L. americanus* according to ref. ^55^] in Scandinavia is in the basal part of the *Peltura* Superzone^56–58^ and which results in an age for the base of Stage 10 at 491.1 ± 0.7 Ma if defined at this level. Alternatively, the FAD of *E. notchpeakensis* just slightly below the onset of HERB carbon isotope event has been proposed as the marker for the base of Stage 10^52,53^ (regarding naming of this event, here provisionally referred to as the HERB/Top of Cambrian Excursion (TOCE) excursion, see the section below). In Scandinavia, this index fossil has been recorded from the *Parabolina lobata* Zone^59^. If defined at this level, the age of the base of Stage 10 is 488.6 ± 0.8 Ma (Fig. 4). This date is consistent with the date calculated for the globally recognized HERB/TOCE event at ~488.0 Ma according to the calibrated 405-kyr framework (Fig. 5).

### The Baltoscandian astrochronology in a global context

The current study provides new and significantly more detailed temporal constraints on the palaeoenvironmental and biological changes during the late Cambrian than previously published (Fig. 5). The globally recognized Steptoean Positive Carbon Isotope Excursion (SPICE) represents the largest carbon cycle perturbation during the late Cambrian, and it is one of the best characterized anoxic events in the pre-Mesozoic ocean^2,3^. Based on the age model (Fig. 5), this excursion in the Baltoscandian Alum Shale started at ~497.5 Ma slightly prior to the Guzhangian–Paibian boundary and lasted for ~3.0 Myr before returning to pre-excursion values. This duration is in line with the previous estimate of 3.0 ± 0.2 Myr^27^. A robust positive carbon isotope excursion (post-SPICE in Fig. 5) centres at ~494 Ma with a duration of ~1.2 Myr. This excursion is similar in position and magnitude to a reported positive δ^13^C feature with an amplitude of up to 2‰ immediately above the SPICE event in carbonate successions from Siberia, Kazakhstan and Laurentia^60,61^, as well as shales from Avalonia and Baltica^8,62^. The HERB, by some referred to as the TOCE, has been recognized in Laurentia, Gondwana, China and Baltica^8,52,53,63–67^. However, whether HERB and TOCE are synonymous remains contentious^68,69^, and for this reason, we provisionally refer to the excursion as HERB/TOCE. Regardless of its name, our astrochronologic framework suggests that this excursion peaked at ~488.0 Ma (Fig. 5). The refined temporal framework serves to clarify the timing and relationship between the major late Cambrian carbon cycle perturbations, environmental changes, and biotic turnovers.

During the latest Miaolingian–earliest Furongian, loss of richness among shelf faunas has been reported from both south-western marginal Laurentia^70–72^, South China^7^, as well as in global compilations^5^. The event is known as the end-Marjuman extinctions, and it partially overlaps with the onset of the SPICE event^73^. Our temporal compilation of the rates of biotic and abiotic events (Fig. 5) shows that as extinctions peaked during the latest Miaolingian, sea water temperatures and sea level were both at their late Cambrian maximum. Late Cambrian–Early Ordovician extreme heat has been suggested by, for instance, clumped isotope evidence^74,75^. This “hyperwarming” is proposed resulting from increasing global insolation due to major eustatic rise and marine onlap of cratons, which probably reduced ocean circulation, lowered oceanic oxygen solubility and promoted epeiric sea anoxia^76^. This extreme warming interval straddles four 405-kyr cycles (E34–E37) of the *Agnostus pisiformis* Zone in Baltica. Hereafter, twelve 405-kyr cycles follow from E33 to E22 that encompass the Paibian *Olenus* Superzone and Jiangshanian *Parabolina* Superzone before a rapid burst in generic richness occurred during the short *Leptoplastus* Superzone spanning only 1.3 405-kyr cycles (i.e., ~500 kyr). This rapid rebound appears to have occurred at least ~4.8 Myr after the extinctions and coincides with isotopic evidence for a dramatic ocean cooling in the palaeo-tropics to temperatures similar to the modern equatorial range^74^ (Fig. 5). The richness burst peaked during the *Protopeltura* Superzone and coincided with a sea-level rise (Fig. 5).

This calibrated 405-kyr framework thus presents a first step towards establishing a well-constrained temporal perspective on the late Cambrian world. Further investigations are needed to understand why the rates of biotic turnover, as well as the environmental determinants, apparently fluctuated so rapidly during the studied interval.

## Methods

### XRF-core scanning

Albjära-1 is a fully cored shallow scientific well with a total depth of 237.40 m made by the University of Copenhagen and the Geological Survey of Denmark and Greenland. The drill site is located about 5 km NE of the small town Svalöv in Scania, Sweden, and the approximate coordinates are 55°56'9.03“N 13°10'42.52“E. The core diameter is 55 mm and the core recovery was essentially 100%. The Alum Shale Formation was penetrated between 135.12 and 232.50 m below ground level at the drill site. The bulk elemental composition of the upper part of the Alum Shale in the Albjära-1 core (135.12–212.49 m) was measured at the GLOBE Institute, University of Copenhagen, using an Itrax X-ray fluorescence core scanner (XRF-CS) equipped with a rhodium tube as the X-ray source. The measurements were conducted on the outer, round, cleaned core surface at a stratigraphic resolution of 1 mm (corresponding to ~200 years of sedimentation on average), confidently recording all expected astronomical cycles. Each scan lasted for 10 s with the voltage and current of Rh energy of 40 kv and 10 mA, respectively. The recorded XRF signals were then analysed with Q-Spec software CoreScanner 8.6.4 Rh from Cox Analytical Systems to get the elemental concentrations using the SGR-1 calibration standard (Green River Shale).

### Handheld-XRF

For broken core intervals (accounting for ~2.5 m of the ~77-m-long core) where XRF-CS was impossible to apply, the element concentrations were measured at the Geological Survey of Denmark and Greenland using a handheld Niton^TM^ Xl3t Goldd+XRF device (HH-XRF) equipped with an Ag anode. Each measurement lasted for 120 s at a 30 kv voltage and 200 μA current. The scanned area is about 5 mm in diameter. In total, 175 shale samples were measured, including 30 from two unbroken intervals (166.25–166.42 and 166.53–166.72 m) for calibration with the XRF-CS concentration (Supplementary Fig. 2), and 145 from broken intervals. The Al concentrations measured by the HH-XRF and XRF-CS methods show a good correlation with Pearson correlation coefficient (*r*^2^) values of 0.93 (Supplementary Fig. 2). Based on the fitted curve, the HH-XRF data were corrected and a complete Al series along the entire length of the core was obtained.

### Organic carbon isotopes

366 Alum Shale powder samples devoid of visible macroscopic pyrite concretions, calcite veins and limestone intercalations were collected using a low-speed micro-drill across the studied interval in the Albjära-1 and Gislövshammar-2 cores. Appropriate amounts (~15 mg) of powder were loaded into open silver-foil capsules. The samples were decarbonated in a vacuum desiccator (5 L) by using concentrated (12 M) hydrochloric acid fumigation for 48 h. Subsequently, the carbonate-free residue was rinsed with deionized water to get a nearly neutral pH. After drying at a temperature of 45°C for 4 h, the samples with silver-foil capsules were transferred to tin combustion cups and closed. The stable carbon isotope analysis was performed at the University of Copenhagen, using an elemental analyzer (CE1110, Thermo Electron, Milan, Italy) connected to an isotope ratio mass spectrometer (IRMS; Finnigan MAT Delta PLUS, Thermo Scientific, Bremen, Germany). The analytical precision was maintained at ±0.08‰ (SD) based on replicated analyses of certified reference material of loamy soil (calibrated by Elemental Microanalysis, Okehampton, UK). All data are reported in the delta notation (δ^13^C_org_) relative to the international standard Vienna Pee Dee Belemnite.

### Thickness correction

The Alum Shale Formation contains abundant lenticular limestone nodules up to about 1 m thick (Fig. 1c and Supplementary Fig. 3a). They are early diagenetic concretions composed of calcite, clay and pyrite^77^. The total accumulated thickness of these nodules in the studied interval of the Albjära-1 well is ~4.2 m. The limestones formed due to a complex interplay of variations in sedimentation rate and availability of various elements that favoured the growth of concretions immediately below the sea floor prior to compaction. They represent a mixing of primary depositional and diagenetic signals. Simply removing all limestone nodules from the data will mistakenly delete certain depositional periods, corresponding to the laterally equivalent shales. Here, we introduce a model to optimize the thickness correction (Supplementary Fig. 3b). The lime content of the nodules is typically c. 80%^77^. We assume that shale mud and minor organic matter account for the remainder 20%. The 80% volume originally occupied by water in the newly deposited clay has disappeared in the shale as the pore water was squeezed out, while the pore volume in the limestone concretions was occupied by early precipitated lime and could not be compacted. Consequently, for every limestone interval, we reduced the thickness to 20%, corresponding to the compacted thickness of the original clay framework. Depths indicated for the Albjära-1 core are adjusted by assigning the Alum Shale Formation top at 135.12 m (drilled depth) as the starting point and measuring downward after reducing the limestone thickness to 20%. Supplementary Table 1 provides a list of adjusted versus original depths.

### Time series methods

The uncalibrated elemental data were first smoothed every 12 measuring points, corresponding to ~2–3 kyr temporal resolution (12 mm). This procedure enhances the signal to noise with minimal risk of overlooking Milankovitch cycles^27^, and translates into a longer exposure time (120 s) sufficient for semi-quantitative analysis of detectable elements^78^. Long-term trends in the uncalibrated Al data were removed by subtracting an 8% and 35–80% weighted average (LOESS) from the data series of the entire and four subsets (0–18, 18–41, 41–55, 55–74 m), respectively. Power spectral analysis was performed using the MTM^79^, with confidence levels of 90%, 95%, 99% and 99.9% calculated from a robust AR(1) noise model^80^. The power decomposition method^81^ was applied to subtract power/variance. Evolutive spectrograms were produced using the evoFFT, to identify frequency changes due to sedimentation rate variations^82^. By identifying long orbital eccentricity cycle nodes and defining equal time spans of 405 kyr between every two minima, the sedimentation rate was calculated and compared to the results of the eCOCO function^83^. All cyclostratigraphic tools are from the software Acycle 2.1 for cyclostratigraphy^84^ except for the Taner filter used for isolating potential astronomical parameters (the script of the latter is shared by Linda Hinnov at http://mason.gmu.edu/~lhinnov/cyclotools/tanerfilter.m). Further details of data handling are presented in Supplementary Note 3.

## Supplementary information

Supplementary Information

## Data Availability

The geochemical data used in this study are provided in the Supplementary Data file. Source Data are provided with this paper.

## Source data

Source Data

## References

[1] Saltzman MR (2011). Pulse of atmospheric oxygen during the late Cambrian. Proc. Natl Acad. Sci. USA..

[2] Gill BC (2011). Geochemical evidence for widespread euxinia in the Later Cambrian ocean. Nature.

[3] Dahl TW (2014). Uranium isotopes distinguish two geochemically distinct stages during the later Cambrian SPICE event. Earth Planet. Sci. Lett..

[4] Saltzman MR, Edwards CT, Adrain JM, Westrop SR (2015). Persistent oceanic anoxia and elevated extinction rates separate the Cambrian and Ordovician radiations. Geology.

[5] Rasmussen CMØ, Kröger B, Nielsen ML, Colmenar J (2019). Cascading trend of Early Paleozoic marine radiations paused by Late Ordovician extinctions. Proc. Natl Acad. Sci. USA..

[6] Fan JX (2020). A high-resolution summary of Cambrian to Early Triassic marine invertebrate biodiversity. Science.

[7] Zhang, S. H., Fan, J. X., Morgan, C. A., Henderson, C. M. & Shen, S. Z. Quantifying the middle–late Cambrian trilobite diversity pattern in South China. *Palaeogeogr. Palaeoclimatol. Palaeoecol*. **570**, 110361 (2021).

[8] Zhao, Z. et al. High-resolution carbon isotope chemostratigraphy of the middle Cambrian to lowermost Ordovician in southern Scandinavia: implications for global correlation. *Glob. Planet. Change* **209**, 103751 (2022).

[9] Peng, S. C., Babcock, L. E. & Ahlberg, P. The Cambrian Period. in *Geologic Time Scale 2020* (eds Gradstein, F. M., Ogg, J. G., Schmitz, M. D., & Ogg, G. M.) 565–629 (Elsevier, Amsterdam, Netherlands, 2020).

[10] Cooper, R. A., Sadler, P. M., Hammer, O. & Gradstein, F. M. The Ordovician Period. in *Geologic Time Scale 2012* (eds Gradstein, F. M., Ogg, J. G., Schmitz, M. D., & Ogg, G. M.) 489–523 (Elsevier, Amsterdam, Netherlands, 2012).

[11] Landing E (2000). Cambrian–Ordovician boundary age and duration of the lowest Ordovician Tremadoc Series based on U–Pb zircon dates from Avalonian Wales. Geol. Mag..

[12] Hinnov LA (2013). Cyclostratigraphy and its revolutionizing applications in the earth and planetary sciences. Geol. Soc. Am. Bull..

[13] Hinnov, L. A. Cyclostratigraphy and astrochronology In 2018. in *Stratigraphy and Time Scales* Vol. 3 (ed Montenari, M.) 1–80 (Academic Press, Amsterdam, 2018).

[14] Berger A, Loutre MF (2004). Astronomical theory of climate change. J. de. Phys. IV (Proc.).

[15] Wu HC (2013). Time-calibrated Milankovitch cycles for the late Permian. Nat. Commun..

[16] Hinnov, L. A. & Hilgen, F. J. Cyclostratigraphy and astrochronology. In *Geologic Time Scale 2012* (eds Gradstein, F. M., Ogg, J. G., Schmitz, M., & Ogg, G.) 63–83 (Elsevier, Amsterdam, Netherlands, 2012).

[17] Laskar J (2004). A long-term numerical solution for the insolation quantities of the Earth. Astron. Astrophys..

[18] Laskar, J., Fienga, A., Gastineau, M. & Manche, H. La2010: a new orbital solution for the long-term motion of the Earth. *Astron. Astrophys*. **532**, A89 (2011).

[19] Berger A, Loutre MF, Laskar J (1992). Stability of the astronomical frequencies over the earths history for paleoclimate studies. Science.

[20] Zhang SC (2015). Orbital forcing of climate 1.4 billion years ago. Proc. Natl Acad. Sci. USA..

[21] Meyers SR, Malinverno A (2018). Proterozoic Milankovitch cycles and the history of the solar system. Proc. Natl Acad. Sci. USA..

[22] Lantink ML, Davies J, Mason PRD, Schaltegger U, Hilgen FJ (2019). Climate control on banded iron formations linked to orbital eccentricity. Nat. Geosci..

[23] Osleger DA, Read JF (1991). Relation of eustasy to stacking patterns of meter-scale carbonate cycles, Late Cambrian, USA. J. Sediment. Petrol..

[24] Bond GC, Devlin WJ, Kominz MA, Beavan J, Mcmanus J (1993). Evidence of astronomical forcing of the earths climate in Cretaceous and Cambrian Times. Tectonophysics.

[25] Bazykin, D. A. & Hinnov, L. A. Orbitally-driven depositional cyclicity of the Lower Paleozoic Aisha-Bibi seamount (Malyi Karatau, Kazakstan): integrated sedimentological and time series study. In *Paleozoic Carbonates of the Commonwealth of Independent States (CIS): Subsurface Reservoirs and Outcrop Analogs* (eds Zempolich, W. G. & Cook, H. E.) 19–41 (Society for Sedimentary Geology, 2002).

[26] Fang JC (2020). Cyclostratigraphy of the global stratotype section and point (GSSP) of the basal Guzhangian Stage of the Cambrian Period. Palaeogeogr. Palaeoclimatol. Palaeoecol..

[27] Sørensen AL (2020). Astronomically forced climate change in the late Cambrian. Earth Planet. Sci. Lett..

[28] Landing E, Bowring SA, Fortey RA, Davidek KL (1997). U–Pb zircon date from Avalonian Cape Breton Island and geochronologic calibration of the early Ordovician. Can. J. Earth Sci..

[29] Davidek K (1998). New uppermost Cambrian U–Pb date from Avalonian Wales and age of the Cambrian–Ordovician boundary. Geol. Mag..

[30] Nielsen AT, Schovsbo NH (2011). The Lower Cambrian of Scandinavia: depositional environment, sequence stratigraphy and palaeogeography. Earth Sci. Rev..

[31] Nielsen AT, Høyberget M, Ahlberg P (2020). The Furongian (upper Cambrian) Alum Shale of Scandinavia: revision of zonation. Lethaia.

[32] Nielsen AT, Weidner T, Terfelt F, Hoyberget M (2014). Hoyberget, M. Upper Cambrian (Furongian) biostratigraphy in Scandinavia revisited: definition of superzones. GFF.

[33] Nielsen AT, Schovsbo NH, Klitten K, Woollhead D, Rasmussen CM (2018). Gamma-ray log correlation and stratigraphic architecture of the Cambro–Ordovician Alum Shale Formation on Bornholm, Denmark: evidence for differential syndepositional isostasy. Bull. Geol. Soc. Den..

[34] Brumsack HJ (2006). The trace metal content of recent organic carbon-rich sediments: Implications for Cretaceous black shale formation. Palaeogeogr. Palaeoclimatol. Palaeoecol..

[35] Tribovillard N, Algeo TJ, Lyons T, Riboulleau A (2006). Trace metals as paleoredox and paleoproductivity proxies: an update. Chem. Geol..

[36] Waltham D (2015). Milankovitch period uncertainties and their impact on cyclostratigraphy. J. Sediment. Res..

[37] Schovsbo NH (2003). The geochemistry of Lower Palaeozoic sediments deposited on the margins of Baltica. Bull. Geol. Soc. Den..

[38] Torsvik, T. H. & Cocks, L. R. M. Cambrian. In *Earth History and Palaeogeography* (eds Torsvik, T. H. & Cocks, L. R. M.) 85–100 (Cambridge Univ. Press, Cambridge, 2016).

[39] Boulila S (2011). On the origin of Cenozoic and Mesozoic “third-order” eustatic sequences. Earth Sci. Rev..

[40] Raiswell R, Canfield DE (2012). The iron biogeochemical cycle past and present. Geochem. Perspect..

[41] Cooper RA, Nowlan GS, Williams SH (2001). Global stratotype section and point for base of the Ordovician system. Episodes.

[42] Azmy K, Stouge S, Brand U, Bagnoli G, Ripperdan R (2014). High-resolution chemostratigraphy of the Cambrian–Ordovician GSSP: enhanced global correlation tool. Palaeogeogr. Palaeoclimatol. Palaeoecol..

[43] Westergård, A. H. Stratigraphic results of the borings through the Alum Shales of Scania made in 1941–1942. *Lunds Geologiska Fältklubb*, 185–204 (1942).

[44] Westergård, A. H. Borrningar genom Skånes alunskiffer 1941–42. in *Sver. Geol. Unders*. Vol. C459 1–45 (Stockholm, 1944).

[45] Ogg, J. G., Ogg, G. M. & Gradstein, F. M. *A Concise Geologic Time Scale 2016*. 234 (Elsevier, Amsterdam, Netherlands, 2016).

[46] Goldman, D. et al. The Ordovician Period. in *Geologic Time Scale 2020* (eds Gradstein, F. M., Ogg,J. G., Schmitz, M. D., & Ogg, G. M.) 631–694 (Elsevier, Amsterdam, Netherlands, 2020).

[47] Schmitz, M. D. Radioisotopic ages used in GTS2020. In *Geologic Time Scale 2020* (eds Gradstein, F. M., Ogg, J. G., Schmitz, M. D., & Ogg, G. M.) 1285–1349 (Elsevier, Amsterdam, Netherlands, 2020).

[48] Henningsmoen, G. The trilobite family Olenidae with description of Norwegian material and remarks on the Olenid and Tremadocian Series. In *Skrifter utgitt av Det Norske Videnskaps-Akademi i Oslo, I. Matematisk-Naturvidenskapelig Klasse* Vol. 1957, 1–303 (1957).

[49] Gradstein, F. M., Ogg, J. G., Schmitz, M. D. & Ogg, G. M. *Geologic Time Scale 2020*. 1357 (Elsevier, Amsterdam, Netherlands, 2020).

[50] Babcock LE, Peng SC, Geyef G, Shergold JH (2005). Changing perspectives on Cambrian chronostratigraphy and progress toward subdivision of the Cambrian system. Geosci. J..

[51] Peng, S. C. et al. Intraspecific variation and taphonomic alteration in the Cambrian (Furongian) agnostoid *Lotagnostus americanus*: new information from China. *Bull. Geosci*. **90**, 281–306 (2015).

[52] Landing, E., Westrop, S. R. & Miller, J. F. Globally practical base for the uppermost Cambrian (Stage 10): FAD of the conodont *Eoconodontus notchpeakensis* and the Lawsonian Stage. In *The 15th Field Conference of the Cambrian Stage Subdivision Working Group, International Subcommission on Cambrian Stratigraphy* (eds Fatka, O. & Budil, P.) 18 (Czech Geological Survey, Prague, 2010).

[53] Landing, E., Westrop, S. R. & Adrain, J. M. The Lawsonian Stage – the *Eoconodontus notchpeakensis* FAD and HERB carbon isotope excursion define a globally correlatable terminal Cambrian stage. *Bull. Geosci*. **86**, 621–640 (2011).

[54] Peng, S. C., Babcock, L. E. & Cooper, R. A. The Cambrian Period. in *Geologic Time Scale 2012* (eds Gradstein, F. M., Ogg, J. G., Schmitz, M. D., & Ogg, G. M.) 437–488 (Elsevier, Amsterdam, Netherlands, 2012).

[55] Ahlberg P, Terfelt F (2012). Furongian (Cambrian) agnostoids of Scandinavia and their implications for intercontinental correlation. Geol. Mag..

[56] Westergård, A. H. *Sveriges olenidskiffer*. 1–205 (Sveriges Geologiska Undersökning Ca18, Uppsala, 1922).

[57] Westergård AH (1947). Supplementary notes on the upper Cambrian trilobites of Sweden. Sver. Geologiska UndersöKnin..

[58] Ahlberg P, Ahlgren J (1996). Agnostids from the Upper Cambrian of Västergötland, Sweden. GFF.

[59] Bagnoli G, Stouge S (2014). Upper Furongian (Cambrian) conodonts from the Degerhamn quarry road section, southern Öland, Sweden. GFF.

[60] Saltzman MR (2004). The Late Cambrian SPICE (δ^13^C) event and the Sauk II-Sauk III regression: New evidence from Laurentian basins in Utah, Iowa, and Newfoundland. J. Sediment. Res..

[61] Kouchinsky A (2008). The SPICE carbon isotope excursion in Siberia: a combined study of the upper Middle Cambrian–lowermost Ordovician Kulyumbe River section, northwestern Siberian Platform. Geol. Mag..

[62] Woods MA, Wilby PR, Leng MJ, Rushton AWA, Williams M (2011). The Furongian (late Cambrian) Steptoean Positive Carbon Isotope Excursion (SPICE) in Avalonia. J. Geol. Soc..

[63] Ripperdan RL, Magaritz M, Nicoll RJ, Shergold JH (1992). Simultaneous changes in carbon isotopes, sea level, and conodont biozones within the Cambrian-Ordovician boundary interval at Black Mountain, Australia. Geology.

[64] Miller, J. F., Evans, K. R., Freeman, R. L., Ripperdan, R. L. & Taylor, J. F. Proposed stratotype for the base of the Lawsonian Stage (Cambrian Stage 10) at the First Appearance Datum of *Eoconodontus notchpeakensis* (Miller) in the House Range, Utah, USA. *Bull. Geosci*. 595–620 (2011).

[65] Terfelt F, Eriksson ME, Schmitz B (2014). The Cambrian–Ordovician transition in dysoxic facies in Baltica—diverse faunas and carbon isotope anomalies. Palaeogeogr. Palaeoclimatol. Palaeoecol..

[66] Li DD (2017). High-resolution C-isotope chemostratigraphy of the uppermost Cambrian stage (Stage 10) in South China: implications for defining the base of Stage 10 and palaeoenvironmental change. Geol. Mag..

[67] Ahlberg P (2018). Integrated Cambrian biostratigraphy and carbon isotope chemostratigraphy of the Grönhögen-2015 drill core, Öland, Sweden. Geol. Mag..

[68] Landing E, Ripperdan RL, Geyer G (2020). Uppermost Cambrian carbon chemostratigraphy: the HERB and undocumented TOCE events are not synonymous. Geol. Mag..

[69] Zhu, M. Y., Babcock, L. E., Peng, S. C. & Ahlberg, P. Reply to ‘Uppermost Cambrian carbon chemostratigraphy: the HERB and undocumented TOCE events are not synonymous’. *Geol. Mag*. **158**, 1–4 (2020).

[70] Westrop, S. R. & Ludvigsen, R. Biogeographic control of trilobite mass extinction at an Upper Cambrian “biomere” boundary. in *Paleobiology* Vol 13, 84–99 (1987).

[71] Westrop SR (1988). Trilobite diversity patterns in an Upper Cambrian Stage. Paleobiology.

[72] Sundberg FA (1996). Morphological diversification of Ptychopariida (Trilobita) from the Marjumiid biomere (Middle and Upper Cambrian). Paleobiology.

[73] Gerhardt AM, Gill BC (2016). Elucidating the relationship between the later Cambrian end-Marjuman extinctions and SPICE Event. Palaeogeogr. Palaeoclimatol. Palaeoecol..

[74] Goldberg, S. L., Present, T. M., Finnegan, S. & Bergmann, K. D. A high-resolution record of early Paleozoic climate. *Proc. Natl. Acad. Sci. USA*. **118**, e2013083118 (2021). 10.1073/pnas.2013083118 PMC8017688 33526667

[75] Quinton PC, Speir L, Miller J, Ethington R, Macleod KG (2018). Extreme heat in the early Ordovician. Palaios.

[76] Landing E (2012). Time-specific black mudstones and global hyperwarming on the Cambrian–Ordovician slope and shelf of the Laurentia palaeocontinent. Palaeogeogr. Palaeoclimatol. Palaeoecol..

[77] Buchardt B, Nielsen AT (1985). Carbon an oxygen isotope composition of Cambro-Silurian limestone and anthraconite from Bornholm (Denmark): evidence for deep burial diagenesis. Bull. Geol. Soc. Den..

[78] de Winter NJ, Sinnesael M, Makarona C, Vansteenberge S, Claeys P (2017). Trace element analyses of carbonates using portable and micro-X-ray fluorescence: performance and optimization of measurement parameters and strategies. J. Anal. Spectrom..

[79] Thomson DJ (1982). Spectrum estimation and harmonic-analysis. P Ieee.

[80] Mann ME, Lees JM (1996). Robust estimation of background noise and signal detection in climatic time series. Climatic Change.

[81] Li MS (2016). Obliquity-forced climate during the Early Triassic hothouse in China. Geology.

[82] Kodama, K. P. & Hinnov, L. A. *Rock Magnetic Cyclostratigraphy*. 176 (John Wiley & Sons, West Sussex, UK, 2015).

[83] Li MS, Kump LR, Hinnov LA, Mann ME (2018). Tracking variable sedimentation rates and astronomical forcing in Phanerozoic paleoclimate proxy series with evolutionary correlation coefficients and hypothesis testing. Earth Planet. Sci. Lett..

[84] Li MS, Hinnov LA, Kump LR (2019). Acycle: time-series analysis software for paleoclimate research and education. Comput. Geosci..

[85] Scotese, C. R. *Atlas of Cambrian and Early Ordovician Paleogeographic Maps (Mollweide Projection), Maps 81–88, Volumes 5, The Early Paleozoic, PALEOMAP Atlas for ArcGIS, PALEOMAP Project, Evanston, IL* (2014).

[86] Miller JF, Evans KR, Freeman RL, Ripperdan RL, Taylor JF (2014). The proposed GSSP for the base of Cambrian Stage 10 at the First Appearance Datum of the conodont *Eoconodontus notchpeakensis* (Miller, 1969) in the House Range, Utah, USA. GFF.

[87] Wang XF (2019). Correlating the global Cambrian-Ordovician boundary: precise comparison of the Xiaoyangqiao section, Dayangcha, North China with the Green Point GSSP section, Newfoundland, Canada. Palaeoworld.

[88] Kröger B, Franeck F, Rasmussen CMØ (2019). The evolutionary dynamics of the early Palaeozoic marine biodiversity accumulation. Proc. R. Soc. B.

