# Supplementary material for: Synchronizing rock clocks in the late Cambrian
Source: Nat Commun. 2022 Apr 13;13:1990. doi: 10.1038/s41467-022-29651-4 (PMC9007955; doi:10.1038/s41467-022-29651-4)
Supplement: Supplementary file 1 — Supplementary Information [file 41467_2022_29651_MOESM1_ESM.pdf]

Nature Communications

Supplementary Information for

Synchronizing rock clocks in the late Cambrian

Zhengfu Zhao*, Nicolas Thibault, Tais W. Dahl, Niels H. Schovsbo, Aske L. Sørensen, Christian M.Ø. Rasmussen, Arne T. Nielsen*

*Corresponding authors: Arne T. Nielsen and Zhengfu Zhao (arnet@ign.ku.dk; zhzh@ign.ku.dk)

Supplementary Note 1. Correlation of biozones, uncertainties, and further consideration over reported radioisotopic ages

Extreme turnover of trilobites in the late Cambrian allows establishment of a detailed biozonation, facilitating precise regional correlation. The current scheme for the Furongian of Scandinavia comprises six superzones subdivided into 23 zones[1,2]. The intensively studied, fully cored Gislövshammar-1 and Gislövshammar-2 wells in SE Scania (located approximately 90 km SE of the Albjära-1 well; Fig. 1) constitute the reference section for the Alum Shale Formation[3,4]. The Gislövshammar-2 well was drilled some 100 m south of Gislövshammar-1 and the Alum Shale succession is essentially identical in the two wells. The Gislövshammar-1 core was thoroughly studied by Westergård[5,6], and he established a very detailed biozonation (Supplementary Fig. 1). A down-hole gamma-ray (GR) log was obtained in the Gislövshammar-2 well[4]. By correlating the GR log pattern in the Gislövshammar-2 well with the detailed biozonation established in the Gislövshammar-1 well, precise correlations have been established to Bornholm, Denmark[4]. Correlation of the Albjära-1 core is based on the fossil content, GR pattern and $\delta^{13}C_{org}$ trends (Supplementary Fig. 1). The Albjära-1 core has been kept as intact as possible and no further splitting in the search for fossils has been undertaken. Only fossils incidentally exposed on bedding planes in the core have been registered, providing a crude biostratigraphy that constrain the GR and $\delta^{13}C_{org}$ correlations to the Gislövshammar-1 and -2 wells. Vertical changes in the content of molybdenum have been used as an auxiliary tool for correlation of the Alum Shale Formation between the Scandinavian wells (Fig. 2).

Most biozonal boundaries in the Albjära-1 are tightly correlated to the more detailed biozonations of the Gislövshammar-1 and -2 cores using GR and $\delta^{13}C_{org}$ correlations supplemented with fossils, but identifying the exact location of biozone and stage boundaries in the Albjära-1 core still carries some uncertainties as discussed below (Supplementary Fig. 1).

(1) Trilobites are absent in the upper part of the *Acerocarina* Superzone, but correlation of $\delta^{13}C_{org}$ variations and GR patterns enables us to pinpoint the upper boundary of this superzone within a 0.29-m-thick interval (146.71–147.00 m). Based on the sedimentation rate at 405-kyr scale, this interval corresponds to ~0.05 Myr (i.e., ± 0.03 Myr error if the Cambrian–Ordovician boundary is assumed located at 146.86 m in the middle of that interval, as stated in the main text).

(2) The lower boundary of the *Acerocare ecorne* Zone in the Albjära-1 core is correlated with the declining limb of a GR peak and the rising limb of a minor $\delta^{13}C_{org}$ excursion in a 0.80-m-thick interval at ~150 m, based on correlation with the Gislövshammar-1 and -2 wells. The thickness of 0.80 m at this level equates to ~0.17 Myr (biozone error e_{bio}= ± 0.09 Myr, assuming that the boundary is located in the middle of that interval).

(3) The trilobite-barren interval within the upper part of the *Parabolina heres megalops* Zone in the Gislövshammar-1 and -2 cores is ~3.90 m thick, and can be correlated to a ~2.70-m-thick coeval interval in the Albjära-1 core with constraints from the GR log pattern and last occurrence (henceforth LO) of *P. heres megalops* in the Albjära-1 core. This interval represents a duration of c. 0.29 Myr (e_{bio}= + 0.29 Myr given the entire barren interval below is assigned to the *P. heres megalops* Zone, following established practice[4]).

(4) The lower boundary of the *Parabolina lobata* Zone is located within a 0.81-m-thick interval constrained by the first occurrence (FO) of *Peltura westergaardi* in the Albjära-1 core and GR peak correlations with the other two intensively studied cores. Deposition of this interval is estimated to represent 0.11 Myr (e_{bio}= + 0.11 Myr providing that the lower boundary of the *P. lobata* Zone coincides with the FO of *P. westergaardi*[7]).

(5) The lower boundary of the *Peltura* Superzone is located within a 0.43-m-thick interval constrained by the FO of *Ctenopyge tumida* in the Albjära-1 core and GR peak correlations with the Gislövshammar-1 and -2 wells, which equates to c. 0.07 Myr (e_{bio}= + 0.07 Myr if the lower boundary of the *Peltura* Superzone coincides with the FO of *C. tumida*[2]).

(6) Two different horizons have been considered for the base of Stage 10. The lower horizon is marked by the first appearance datum (FAD) of the agnostoid *Lotagnostus americanus*[8,9], and the upper horizon is proposed defined by the FAD of the euconodont *Eoconodontus notchpeakensis* just below the onset of the Hellnmaria-Red Tops Boundary (HERB) carbon excursion event[10,11]. In Baltoscandia, the base of the *L. americanus* agnostoid Zone has for a period been correlated with the *C. spectabilis* trilobite Zone[12,13], corresponding to the upper part of the *Sphaerophthalmus modestus*–*Sphaerophthalmus angustus* trilobite Zone in the revised zonation scheme recently published[14]. However, reassessment of original occurrence data on *L. trisectus* [= *L. americanus* according to reference[13]] shows that its lowest record in Scandinavia is in the basal part of the *Peltura* Superzone, corresponding to the *P. acutidens*–*C. tumida* zone (cf. references[15-17]; Supplementary Fig. 1). Therefore, if the lower boundary of Stage 10 is correlated to the base of this zone, the biozone uncertainty e_{bio} is + 0.07 Myr (see Case 5 above). The alternative marker *E. notchpeakensis* has been recorded in the *P. lobata* trilobite Zone in Sweden[18]. Constrained by GR correlation with the Gislövshammar-1 and -2 wells and the FO of *P. westergaardi* in the Albjära-1 core, the *P. lobata* Zone is inferred located between 158.20–159.69 m. Deposition of this interval is estimated to represent c. 0.29 Myr. Assuming that the alternative lower boundary of Stage 10 is located in the middle of this zone, the biozone uncertainty e_{bio} is ± 0.14 Myr.

(7) No diagnostic fossils were identified in the combined *Sphaerophthalmus postcurrens–Leptoplastus neglectus* zones. This interval, however, can be bracketed by the LO of *Leptoplastus stenotus* (index fossil for the upper zone of the *Leptoplastus* Superzone[2]) and the FO of *Sphaeropthhalmus flagellifer* (index fossil for the overlying eponymous zone in the *Protopeltura* Superzone[2]) in the Albjära-1 core. The unfossiliferous interval has a thickness of 0.44 m, which corresponds to 0.07 Myr (e_{bio}= – 0.07 Myr in determining the upper boundary of the *Leptoplastus* Superzone and e_{bio}= + 0.07 Myr in determining the lower boundary of the *S. flagellifer* Zone).

(8) The *Parabolina–Leptoplastus* superzone boundary is located within a 0.52-m-thick interval constrained by the LO of *Parabolina spinulosa* and the FO of *Leptoplastus* sp., corresponding to c. 0.09 Myr (e_{bio}= – 0.09 Myr if the boundary coincides with the LO of *P. spinulosa*[2,7], where a limestone intercalation in the Albjära-1 core seemingly interferes with the GR correlation).

(9) The *Olenus–Parabolina* superzone boundary is poorly constrained by trilobite occurrences in all Scanian wells, but must be located below 188.80 m in the Albjära-1 core (FO of *Parabolina brevispina*). The boundary is inferred located on the declining limb of a broad GR peak in the Gislövshammar-1 and -2 wells. The equivalent interval is 1.6 m thick in the Albjära-1 core, corresponding to c. 0.36 Myr (e_{bio}= ± 0.18 Myr assuming that the *Olenus–Parabolina* superzone boundary is located in the middle of that interval). This superzone boundary is taken as conterminous with the Paibian–Jiangshanian stage boundary in Scandinavia[19].

(10) In the Albjära-1 core, the *Agnostus pisiformis* Zone–*Olenus* Superzone boundary is located at 201.44 m as indicated by the FO of *Olenus* sp. (*gibbosus*?) and *Homagnostus obesus*, which in Scandinavia co-occur with the primary marker *Glyptagnostus reticulatus* for the base of Paibian Stage[2,7,12,13,20]. The trilobite-barren interval in the upper part of the *Agnostus pisiformis* Zone in the Gislövshammar-1 and -2 cores has a thickness of 1.00 m, and can be correlated to a 1.21-m-thick interval in the Albjära-1 core with constraints from the GR correlation, the LO of *Agnostus pisiformis* as well as the FO of *Olenus* sp. (*gibbosus*?) and *Homagnostus obesus* in the Albjära-1 core. Deposition of this barren interval lasted about 0.35 Myr (e_{bio}= + 0.35 Myr given that the entire barren interval below the *Olenus* Superzone is routinely assigned to the *A. pisiformis* Zone[4]).

(11) The remaining biozone boundaries are more tightly constrained. Although graptolites are not common within the Tremadocian in the Gislövshammar-1 and -2 cores, the presence of diagnostic fossils in the Albjära-1 core itself define the boundaries between the *Rhabdinopora* spp., *Adelograptus tenellus and Bryograptus kjerulfi* zones. The lower boundaries of the *P. heres megalops* and *Peltura scarabaeoides* zones are both precisely determined using GR correlation. The lower boundary of the *S. modestus–S. angustus* Zone is tightly determined by the LO of *S. flagellifer* and the FO of *S. angustus*(?). The *Parabolina spinulosa* Zone is constrained by GR correlation and the FO of *P. spinulosa*. The base of the Guzhangian Stage (coincident with the base of the *Lejopyge laevigata* Zone) is defined by the FAD of the cosmopolitan agnostoid trilobite *L. laevigata*[21]. In Scandinavia, the lower boundary of the *L. laevigata* Zone is correlated with the base of the Andrarum Limestone, which is a key marker in regional correlation[4,20,22]. For these seven boundaries that are better identified than the other biozone boundaries in the Albjära-1 core, we assign the minimum uncertainty

of 0.07 Myr (e_{bio}= ± 0.07 Myr) obtained above. Regarding the *L. laevigata–A. pisiformis* zonal boundary, its precise location cannot be deduced based on GR correlations or fossil occurrence, and for this reason its absolute age is not provided.

The original U-Pb zircon age of 491± 1 Ma for the volcanic ash at Ogof-ddu, Criccieth, N. Wales[23] was recalculated at 488.71 ± 1.17 by Schmitz[24,25]. However, it is incorrectly updated to 490.1±0.6 Ma in the Cambrian Chapter (p. 613) of the GTS 2020[26] (M.D. Schmitz, pers. commun., 2021). Moreover, since the GTS 2012, there has been so far no reinvestigation of the Bryn-llin-fawr ash bed from the uppermost Furongian *Acerocare ecorne* Zone at Bryn-llin-fawr, North Wales. The original U-Pb age of 489±0.6 Ma reported by Landing et al.[27] was correctly recalculated to 486.78±0.53 Ma by Schmitz[24,25]. Hence, the different age of 490.1±0.57 Ma specified in the Ordovician Chapter (p. 665) of the GTS 2020[28] is erroneous (M.D. Schmitz, pers. commun., 2021).

Supplementary Note 2. Late Cambrian–Early Ordovician astronomical parameters

Unraveling the astronomical parameters is of great importance for the time calibration of the Milankovitch-influenced sedimentary cycles. The 405-kyr long eccentricity cyclicity, caused by gravitational interactions between Jupiter and Venus, is stable and dominant during most of Earth's history due to the great mass of Jupiter[29,30], and is used here for basic calibration of the Cambrian cyclostratigraphy. Short eccentricity periods ranging from 95 to 131 kyr are recognized with an estimated average duration of approximately 100 kyr[29,31,32]. The prediction of obliquity and precession periods has accumulating bias back in time due to the influence from Earth's past tidal dissipation. To tackle this problem, previous researchers assumed a constant lunar-recession rate[31,33] or a constant tidal time lag[29] for estimation of obliquity and precession frequencies. However, these two operations inevitably induce overestimation and underestimation of the Earth–Moon separation, respectively[32]. The latest solution proposed by Waltham[32] estimates the uncertainties in predicted cycle periods, and is adopted in this paper. Consequently, at 492 Ma (the approximate mean age of the Alum Shale interval studied in this paper), a period of 32.7 ± 4.0 kyr is considered for short obliquity, and periods of 20.6 ± 1.6 kyr, 19.6 ± 1.4 kyr, 16.9 ± 1.1 kyr and 17.0 ± 1.1 kyr (average ~18.5 kyr) are considered for precession cycles[32].

Supplementary Note 3. Cyclostratigraphic analysis

3.1 Power spectra analyses in depth domain

The MTM power spectrum of the entire uncalibrated Al data series revealed numerous peaks that generally cluster with wavelengths of 1.79–2.90 m, 0.48–0.76 m, 0.13–0.22 m and 0.08–0.11 m above the 99% confidence level (Supplementary Fig. 4a). When the sedimentation rate (SR) shifts significantly, a cycle can be recorded by multiple frequencies, making it difficult to determine the stratigraphic time scale[34,35]. In this study, the evoFFT spectrum revealed frequency changes at ~18 m, ~41 m and ~55 m (Supplementary Fig. 4b),

which likely result from SR variations linked to sea level changes (high SR during sea level lowstands, low SR during sea level highstands, see fig. 2 in ref.[2], and Supplementary Note 3.2.2 in this study). Therefore, the whole Al series was divided into four subsets for the MTM analysis: 0–18 m, 18–41 m, 41–55 m, and 55–74 m (Supplementary Fig. 4c).

For subset I (0–18 m), the MTM spectrum shows significant wavelength peaks of ~1.85 m, 0.45–0.63 m, 0.14–0.17 m and 0.08–0.11 m, which are approximately comparable with the theoretic periodic ratios of long eccentricity, short eccentricity, obliquity and precession (i.e., 405 kyr, ~100 kyr, ~32.7 kyr, and ~18.5 kyr) for the late Cambrian (see Supplementary Note 2). The average SR (compacted values) for the Furongian interval covering 135.12 m to 201.44 m in the Albjära-1 core is ~5 mm/kyr (using GTS2020 dates), which is close to the ~4 mm/kyr estimate for the entire upper Cambrian Alum Shale of Scania[36]. The calculated thicknesses corresponding to 405 kyr, ~100 kyr, ~32.7 kyr and ~18.5 kyr are 1.62–2.03 m, 0.40–0.50 m, 0.13–0.16 m, and 0.07–0.09 m, respectively. These figures match the above observed wavelengths. Thus, the observed sedimentary cycles of ~1.85 m, 0.43–0.63 m, 0.14–0.17 m, and 0.08–0.11 m likely reflect orbital forcing, and are interpreted to correspond to long eccentricity, short eccentricity, obliquity and precession cycles, respectively (Supplementary Fig. 4c).

For subset II (18–41 m), the MTM power spectrum exhibits obvious peaks at ~2.44 m, ~0.68 m, 0.17–0.20 m and 0.09–0.12 m, fitting very well with the theoretical orbital ratios. The most obvious wavelength of ~2.44 m is inferred to reflect deposition in periods of ~488–610 kyr, based on the cited average accumulation rates of 4–5 mm/kyr. The greater than expected thicknesses are attributed to the circumstance that subset II includes at least two significant sea level lowstand intervals within the *Protopeltura–Leptoplastus* and the upper *Peltura* superzones[2], where the actual SRs may have been higher than the average 4–5 mm/kyr, as shown in Supplementary Fig. 5. Therefore, based on astronomical cycle ratios, it is reasonable to assume that the ~2.44 m cycle reflects the 405-kyr long eccentricity signal and in accordance with this, all other peaks cited above are interpreted as short eccentricity, obliquity and precession cycles, respectively (Supplementary Fig. 4c).

For subset III (41–55 m), the MTM spectrum displays similar wavelengths as subset II. A major sea level lowering during the later part of the *Olenus* Superzone and the entire *Parabolina* Superzone was likely associated with increased sedimentary supply to the outer shelf[2], and the SR may be anticipated to exceed the average value as shown in Supplementary Fig. 5. Consequently, the ~2.78 m, ~0.68 m, ~0.19 m and 0.10–0.14 m cycles are inferred to represent long eccentricity, short eccentricity, obliquity and precession cycles, respectively (Supplementary Fig. 4c).

For subset IV (55–74 m), the MTM spectrum exhibits peaks at ~1.84 m, ~0.49 m, ~0.14 m and 0.08–0.11 m, approaching the wavelengths discussed for subset I. Therefore, we interpret this set of peaks as long eccentricity, short eccentricity, obliquity and precession cycles, respectively (Supplementary Fig. 4c).

Based on the above considerations, we infer that the ~1.85 m, ~2.44 m, ~2.78 m and ~1.84 m wavelengths seen in the four subsets reflect changes in wavelength of the 405-kyr long eccentricity signal which are subsequently filtered out, resulting in 39 cycles for the entire Al series. By identifying the minima

of each 405-kyr cycle, we reconstructed the SR curve through the studied interval at the 405-kyr scale (Supplementary Table 1).

3.2 Test of the calculated sedimentation rate

3.2.1 eCOCO map

The established SR curve is further corroborated by the results of the eCOCO (evolutionary correlation coefficient) analysis (Supplementary Fig. 5), a powerful tool for tracking SR variations[37]. The eCOCO analysis of the entire Al series indicates possible SRs of 1–10 mm/kyr with a correlation coefficient ρ generally above 0.3 (Supplementary Fig. 5b). The evolutionary significance level of the null hypothesis ($H_0\,SL$) displays similar trends with most $H_0\,SL$ values below 0.5% (Supplementary Fig. 5c), and 7 astronomical targets used in eCOCO are investigated in a 1–10 mm/kyr sedimentation rate range (Supplementary Fig. 5d). More importantly, these three spectra are mostly in line with the SR curve interpreted by picking the minima of each 405-kyr cycle (black line in Supplementary Fig. 5).

3.2.2 Sea level changes

The example of biofacies distribution in Scandinavia documented by Nielsen et al.[2] corroborates the resulting SR curve. The Alum Shale Formation was deposited on the outer shelf from about the storm wave base and deeper[38]. During lowering(s) of the sea level, the sea floor on the mid shelf, such as central Sweden, Öland and the autochthon of Jämtland, came within reach of storms, which recycled the unconsolidated mud and created regional stratigraphic gaps in the succession, occasionally associated with conglomerates formed by exhumed limestone nodules[2]. Concurrently, the distal area of the Alum Shale basin (e.g., Scania, Oslo Region) received increased supply of sediment eroded from the inboard segment of the shelf, thus resulting in higher SRs. Conversely, the SRs lowered on the outer shelf during high sea level as much of the sediment was trapped on the mid shelf. Specifically, as seen in Supplementary Fig. 5, the SRs kept decreasing from 74 m to 64 m, which is consistent with the inference that a major sea level rise during the *L. laevigata* and *A. pisiformis* zones displaced the coastline into Russia. At this stage, deposition of the Alum Shale Formation spread across the greater part of Scandinavia and reached as far east as Gotland in the Baltic Sea. Then, an intermediate sea level stance prevailed at the Miaolingian–Furongian transition, i.e., at the boundary between the *A. pisiformis* Zone and the *Olenus* Superzone. At this stage, south central Sweden came within reach of storm waves, causing reworking of the Alum Shale mud and abrupt increasing SR in the Scanian area. The lower *Olenus* Superzone is comparatively condensed and characterized by a transient sea level rise, resulting in a drop of the SR from ~6 mm/kyr to ~3 mm/kyr. Subsequently, the sea level lowered markedly through the late part of the *Olenus* Superzone and reached a lowstand peak in the early *Parabolina* Superzone (the *P. brevispina* Zone), associated with a SR as high as ~7 mm/kyr at ~50 m corrected depth in the Albjära-1 core. Overall, the entire *Parabolina* Superzone was a second order sea level lowstand but seemingly with a short major rise at some stage in the *P. spinulosa* Zone (uppermost *Parabolina* Superzone)[2], as supported by a significant SR decrease and quick increase at ~44 m. The sea level then started rising again from the *Leptoplastus* Superzone to reach a high in the lower half of the *Peltura* Superzone. This also agrees with SR

decreasing from ~6 mm/kyr at ~40 m to 3 mm/kyr at ~32 m. The *P. lobata* and *P. megalops* zones (constituting the upper part of the *Peltura* Superzone) mark a new sea level lowstand which peaked in the latter zone. At this stage, the sea level was as low as in the *P. brevispina* Zone (*Parabolina* Superzone) with a high SR of ~9 mm/kyr at ~16 m corrected depth in the Albjära-1 core. After a transient rise during the lower part of the *Acerocarina* Superzone followed by a minor fall, the sea level rose significantly into the Tremadocian and attained a new high in the *Rhabdinopora flabelliformis* Zone, at which stage deposition of Alum Shale Formation reached its greatest extent with a very low SR of ~3 mm/kyr at ~5 m corrected depth in the Albjära-1 core.

Summing up, our 405-kyr-derived SR curve fits well with the eCOCO results, and also exhibits a robust anti-correlation with the inferred sea-level changes based on biofacies distribution and regional gaps in the inboard Alum Shale succession, which taken all together, provide a solid foundation for tuning of the astronomical cycle signal.

3.3 Orbital tuning

Based on the well-constrained 405-kyr-derived SRs, the Al signals were tuned to the time domain, enabling the construction of a ~16 Myr-long floating astronomical time scale. The MTM spectrum of the calibrated Al series contains significant peaks reflecting the Milankovitch grand cycles (~2.6 Myr, ~1.8 Myr, ~1.3 Myr), long eccentricity (405 kyr), short eccentricity (~108 kyr), obliquity (~30.9 kyr), and precession (17.1–20.9 kyr, average ~19.2 kyr) above the 99.9% confidence level (Supplementary Fig. 6a, b), consistent with the expected late Cambrian–Early Ordovician astronomical parameters (See Supplementary Note 2 for details). Interestingly, the age model derived from the Al series can also be successfully applied to other lithogenic elements such as silicon (Si) and titanium (Ti) (Supplementary Fig. 6d–i), further corroborating the reliability of our interpretation.

3.4 Amplitude modulation analysis and secular resonance between Earth and Mars orbits

Amplitude modulation analysis is another tool to identify an astronomical origin of the observed cyclicity[39]. Laskar[40] identified a secular resonance $(s_4–s_3)–2(g_4–g_3)=0$ between the motions of Earth and Mars, where s_3 and s_4 are the precession of the node of the Earth and Mars, and g_3 and g_4 are the precession of the perihelia of these planets. However, due to the chaotic motions of the planets, this resonance of $(s_4–s_3)–2(g_4–g_3)=0$ can evolve in a rotational state, and even move to a second librational state corresponding to $(s_4–s_3)–(g_4–g_3)=0$[29,41]. At present, $(s_4–s_3)=2(g_4–g_3)$ is manifested as amplitude modulations in Earth's orbital eccentricity (2.4 Myr) and obliquity (1.2 Myr)[42]. However, prior to ~50 Ma, different modulation periodicities were occurring due to chaotic Earth-Mars orbital resonance (e.g., refs.[35,43]). To date, no modulation periodicities for the Cambrian have been reported. The MTM spectra of Al, Si and Ti in this study display a set of significant peaks at ~1.3 Myr, ~1.8 Myr and ~2.6 Myr (Supplementary Fig. 6). Long-term modulations were evaluated by comparing short eccentricity power-to-total power (e/T) for the ~108-kyr cycles and obliquity power-to-total power (O/T) for the ~30.9-kyr cycles. The e/T of our calibrated Al series started from two ~1.8 Myr cycles at ~498 Ma, gradually changing to a ~2 Myr cycle, then to a single ~2.6 Myr at ~493 Ma

before transitioning back to ~1.7 Myr cycling for two repetitions and then to ~2.6 Myr again at 486 Ma (Supplementary Fig. 7a). Importantly, the O/T maintains a ~1.3 Myr periodicity throughout this interval (Supplementary Fig. 7b). The ~1.3 Myr component is comparable to the ~1.3 Myr obliquity modulation detected in the Ordovician[44,45], and is a bit longer than the ~1.0–1.1 Myr obliquity modulation cycles that have been reported from the late Palaeozoic[46-48]. Our data thus seem to provide geological evidence for Cambrian chaotic resonance transitions associated with interactions between the Earth and Mars.

Supplementary Note 4. Detailed cyclostratigraphic analysis for selected intervals

The 405-kyr periodic variations identified in the Albjära-1 core overall show excellent match with published interpretations of overlapping intervals[49], but minor discrepancies still exist regarding the E25, E34–E35 and E37 cycles, as discussed in the main text. Therefore, we conducted supplementary detailed cyclostratigraphic analyses for these intervals, viz. 47.0–52.0 m, 63.5–67.5 m, and 68.0–73.7 m (corrected depths in the Albjära-1 core). As shown in Supplementary Fig. 8, the well-expressed 405-kyr cycles, associated with higher-frequency oscillations, corroborate the reliability of the presented Aluminium-based interpretations for the Albjära-1 core. For details, see the main text.

Supplementary Note 5. Duration of the Cambrian biozones in Scandinavia

Based on the cyclostratigraphic analysis of the Albjära-1 section, the duration of the Furongian *Olenus*, *Parabolina*, *Leptoplastus*, *Protopeltura*, *Peltura* and *Acerocarina* superzones are calculated at ~3.2 Myr, ~1.6 Myr, ~0.5 Myr, ~0.7 Myr, ~3.5 Myr, ~0.8 Myr, respectively. The calculated duration of the *Olenus* Superzone is comparable with the previous estimate of 3.4 ± 0.2 Myr based on cyclostratigraphic analysis of drill cores from eastern Scania (Sweden) and Bornholm (Denmark)[49]. The duration of the *Parabolina* Superzone is ~0.3 Myr shorter according to our calculations than the previous estimate at 1.9 ± 0.3 Myr, possibly due to a hiatus or condensation linked to a significant sea level lowstand in the lower part of this superzone (as explained in the main text). The *Parabolina* Superzone duration is thus set as the average (~1.8 Myr) of the two estimates with an additional uncertainty of a half 405 kyr, as discussed in the main text (Fig. 4). In addition, our *Leptoplastus* Superzone (~0.5 Myr) and *Protopeltura* Superzone (~0.7 Myr) estimates are slightly longer than those based solely on analysis of the strongly condensed Bornholm section (0.33 ± 0.18 and 0.51 ± 0.20 Myr, respectively[49]). We attribute these differences to the local lack of the *L. paucisegmentatus*, *L. raphidophorus* and *S. postcurrens* zones within the *Leptoplastus* and *Protopeltura* superzones on Bornholm[50]. These hiatuses result from local uplift[4], while the succession is complete in the studied Albjära-1 core from Scania.

Supplementary Tables and Figures

Supplementary Table 1 Assigned stratigraphic depths, calculated sedimentation rates and corresponding floating astronomical time of the 405-kyr eccentricity minima in the Albjära-1 core.

Sys.	Ser.	Stage	Superzone	405-kyr eccentricity cycle	405-kyr eccentricity minima: Original depth (m)	405-kyr eccentricity minima: Adjusted depth (m)[c]	Sedimentation rate (mm/kyr)	Floating astronomical time (Ma)
Ordovician	Lower	Trema-docian	Not defined		135.120	0.000	4.2	0.0
				E1	136.736	1.476	4.2	0.4
				E2	138.551	3.183	3.4	0.8
				E3	139.981	4.562	3.1	1.2
				E4	141.505	5.829	3.6	1.6
				E5	142.948	7.272	4.3	2.0
				E6	144.692	9.016	3.5	2.4
				E7	146.124	10.448	5.6	2.8
Cambrian	Furongian	Stage 10[b]; Stage 10[a]	*Acerocarina*	E8	148.386	12.710	4.6	3.2
				E9	150.285	14.579	9.3	3.6
			Peltura	E10	154.264	18.356	7.9	4.0
				E11	157.482	21.574	4.9	4.4
				E12	159.488	23.576	7.3	4.8
				E13	162.457	26.545	5.2	5.2
				E14	165.088	28.667	4.1	5.6
				E15	166.769	30.341	5.2	6.0
				E16	168.928	32.434	3.3	6.4
				E17	170.254	33.756	4.8	6.8
		Jiangsh-anian	*Protopeltura*	E18	172.465	35.683	6.6	7.2
				E19	175.507	38.366	6.4	7.6
			Leptoplastus	E20	178.226	40.941	5.8	8.0
			Parabolina	E21	180.616	43.277	2.5	8.5
				E22	181.720	44.283	6.4	8.9
				E23	184.554	46.858	5.4	9.3
				E24	186.899	49.065	6.9	9.7
				E25	189.695	51.858	3.7	10.1
		Paibian	*Olenus*	E26	191.189	53.351	4.0	10.5
				E27	192.814	54.977	3.8	10.9
				E28	194.339	56.501	3.2	11.3
				E29	195.630	57.792	3.2	11.7
				E30	196.923	59.084	3.0	12.1
				E31	198.124	60.285	2.9	12.5
				E32	199.330	61.450	6.0	12.9
	Miaolingian	Guzhan-gian	*Paradoxides forchhammeri*	E33	202.107	63.881	2.2	13.3
				E34	202.984	64.758	3.6	13.7
				E35	204.669	66.201	3.5	14.1
				E36	206.346	67.633	5.1	14.5
				E37	208.403	69.690	3.6	14.9
				E38	209.880	71.168	5.0	15.3
				E39	211.923	73.204	5.0	15.7
					212.489	73.720	5.0	15.8

[a] If the base of Stage 10 is defined by the FAD of the conodont *Eoconodontus notchpeakensis* below the onset of Hellnmaria-Red Tops Boundary (HERB) carbon excursion event.
[b] If the base of Stage 10 is defined by the FAD of the agnostoid *Lotagnostus americanus*.
[c] Depth below top of the Alum Shale Formation after reducing the limestone thickness to 20%.

Supplementary Table 2 Chrono- and biostratigraphy of the studied strata in the Albjära-1 well and radioisotopically anchored astronomical time scale. Dashed lines represent uncertain biozone / stage boundaries.

Sys.	Ser.	Stage	Superzone	Zone	Original depth (m)	Adjusted depth (m)[c]	Estimated Time (Ma)[d]
Ordovician	Lower	Trema-docian	Not defined	*Bryograptus kjerulfi*	← 135.12[e]	0.00	483.9±0.7
					137.80	2.46	484.5±0.7
				Adelograptus tenellus			
					139.20	3.83	484.8±0.7
				Rhabdinopora spp.			
					146.86	11.18	486.78±0.56
Cambrian	Furongian	Stage 10[a] / Stage 10[b]	*Acerocarina*	*Acerocare ecorne*			
					149.85	14.17	487.4±0.8
				Westergaardia scanica			
				Acerocarina granulata –Peltura costata			
					151.65	15.74	487.6+1.0/-0.7
			Peltura	*Parabolina h. megalops*			
					158.20	22.29	488.4±0.7
				Parabolina lobata	158.95	23.04	488.6±0.8
					159.69	23.78	488.7+0.8/-0.7
				Peltura scarabaeoides			
					170.35	33.85	490.7±0.7
				Peltura acutidens– Ctenopyge tumida			
					172.42	35.64	491.1±0.7
		Jiangsh -anian	*Protopeltura*	*Sphaeroph. modestus– Sphaeroph. angustus*			
					174.48	37.46	491.4±0.7
				Sphaeroph. flagellifer			
					176.90	39.66	491.7±0.7
				Sphaeroph. postcurrens– Leptoplastus neglectus			
					177.34	40.06	491.8±0.7
			Leptoplastus	*Lepto. stenotus*			
				Lepto. crassicornis– Lepto. angustatus			
				Lepto. raphidophorus			
				Lepto. paucisegmentatus			
					180.33	42.99	492.3+0.7/-0.8
			Parabolina	*Parabolina spinulosa*			
					186.88	49.05	493.5±0.7
				Parabolina brevispina			
					189.70	51.86	494.1±1.0
		Paibian	*Olenus*	*O. scanicus-O. rotundatus*			
				Olenus dentatus			
				Olenus attenuatus			
				Olenus wahlenbergi			
				Olenus truncatus			
				Olenus gibbosus			
					201.44	63.27	497.3+1.2/-0.9
	Miaolingian	Guzhan-gian	*Paradoxides forchhammeri*	*Agnostus pisiformis*			
					← 212.49[f]	73.72	499.9±0.9
				Lejopyge laevigata			
					215.45		500.3±0.9[g]

[a] If the base of Stage 10 is defined by the FAD of the conodont *Eoconodontus notchpeakensis* below the onset of Hellnmaria-Red Tops Boundary (HERB) carbon excursion event..

[b] If the base of Stage 10 is defined by the FAD of the agnostoid *Lotagnostus americanus*.

[c] Depth below top of the Alum Shale Formation after reducing the limestone thickness to 20%.

[d] The uncertainties include the errors of U-Pb ages and carbon isotope constraints on the Cambrian-Ordovician boundary (± 0.56 Myr), the uncertainty of peak assignments in the cyclostratigraphic signal due to nonlinear climatic response that caused variable time lags between orbital forcing and sedimentation cyclic expression (± 0.10 Myr), one 405-kyr discrepancy between this study and that of Sørensen et al.[44] in the *Parabolina* Superzone (± 0.20 Myr), and/or the error in identifying the exact location of biozone or stage boundaries (0.07–0.35 Myr). See text for details.

[e] The top of the Alum Shale Formation in the Albjära-1 core.

[f] The elemental analysis in the Albjära-1 core stopped at 212.49 m within the Alum Shale Formation.

[g] The age of the base of the Guzhangian Stage at 215.45 m was estimated based on the nearest sedimentation rate at 212.49 m.

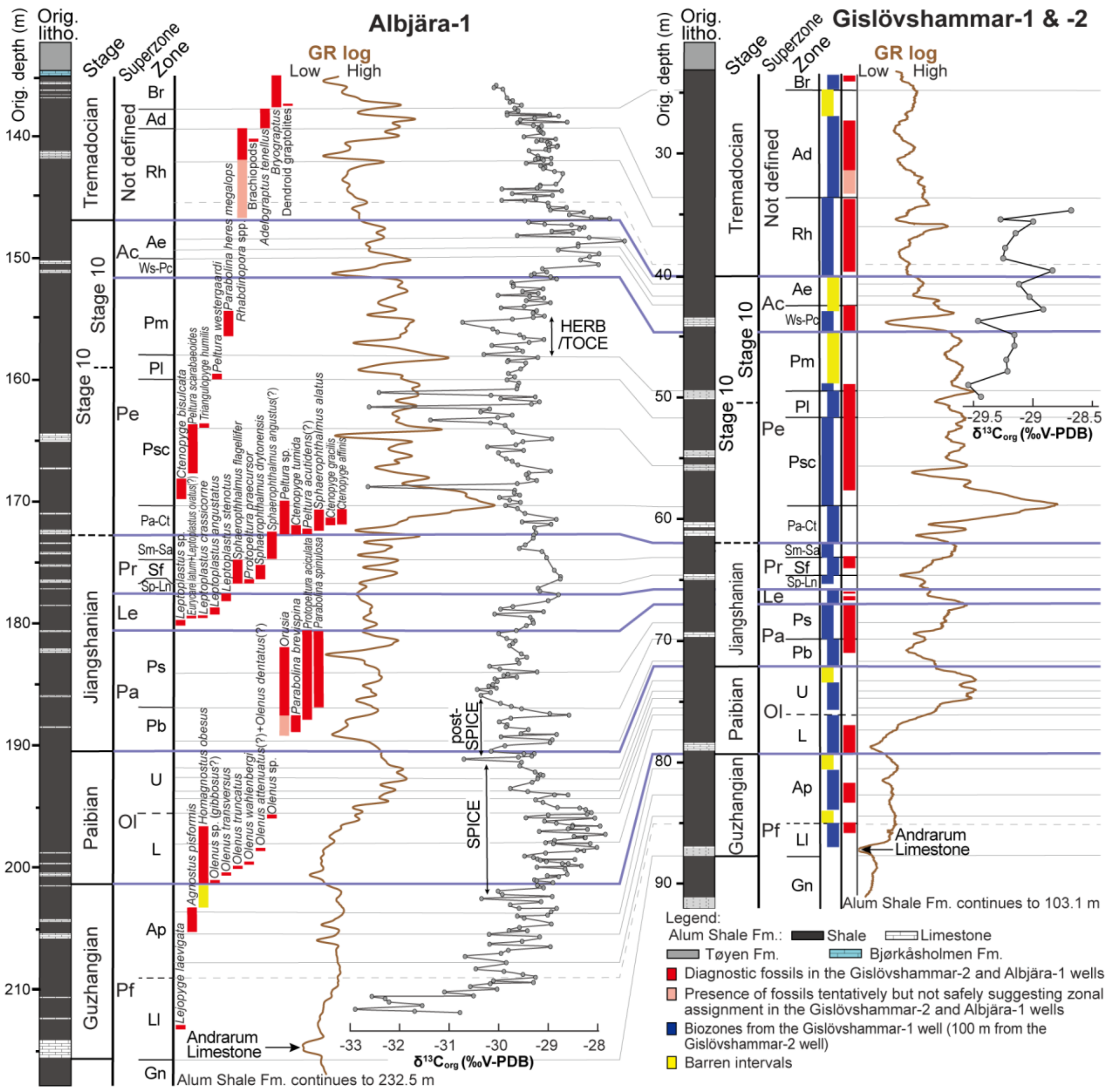

Supplementary Fig. 1 Lithology and biozonation of studied wells (modified from ref.[51]). Correlation of the Alum Shale in the Albjära-1 well with the Gislövshammar-1 and -2 wells in southeastern Scania, based on GR logs, characteristic fossils and carbon isotope trends. The detailed biozonation of the Gislövshammar-1 well is from refs.[5,6], the biozonation and GR curve of the adjacent Gislövshammar-2 well is from ref.[4]. The depth intervals of stages / superzones / zones are shown in Supplementary Table 2. Abbreviations: HERB/TOCE= the Hellnmaria-Red Tops Boundary carbon excursion event / the Top of Cambrian Excursion, SPICE= Steptoean Positive Carbon Isotope Excursion, post-SPICE= post-Steptoean Positive Carbon Isotope Excursion. Tre=Tremadocian, Ac=*Acerocarina*, Pe=*Peltura*, Pr=*Protopeltura*, Le=*Leptoplastus*, Pa=*Parabolina*, Ol=*Olenus*, Pf=*Paradoxides forchhammeri*, Pp=*Paradoxides paradoxissimus*, Br=*Bryograptus kjerulfi*, Ad=*Adelograptus tenellus*, Rh=*Rhabdinopora* spp., Ae=*Acerocare ecorne*, Ws–Pc= *Westergaardia scanica*, *Acerocarina granulata* and *Peltura costata*, Pm=*Parabolina heres megalops*, Pl=*Parabolina lobata*, Psc=*Peltura scarabaeoides*, Ct=*Ctenopyge tumida*, Sm–Sa=*Sphaerophthalmus modestus*–*Sphaerophthalmus angustus*, Sf=*Sphaerophthalmus flagellifer*, Sp–Ln=*Sphaerophthalmus postcurrens*–*Leptoplastus neglectus*, Ps=*Parabolina spinulosa*, Pb=*Parabolina brevispina*, Ap= *Agnostus pisiformis*, Ll=*Lejopyge laevigata*, Gn=*Goniagnostus mathorsti*, L=Lower, U=Upper.

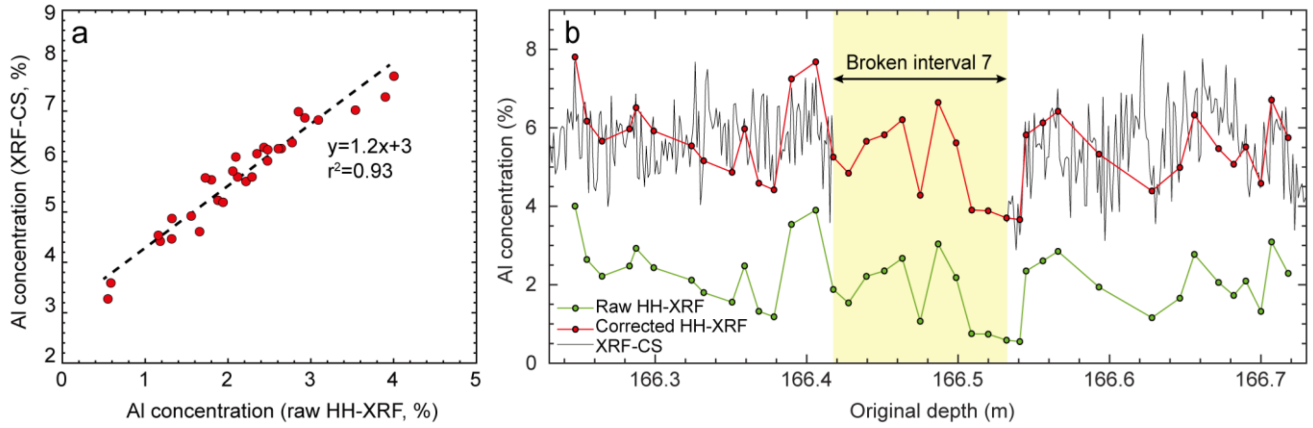

Supplementary Fig. 2 Correction for HH-XRF Al concentrations. **a** Aluminium concentrations determined from HH-XRF and XRF-CS, showing excellent correlation (correlation coefficient r^2 of 0.93). **b** The relationship between the Al concentrations from HH-XRF, XRF-CS and corrected HH-XRF calculated using the function shown in panel a.

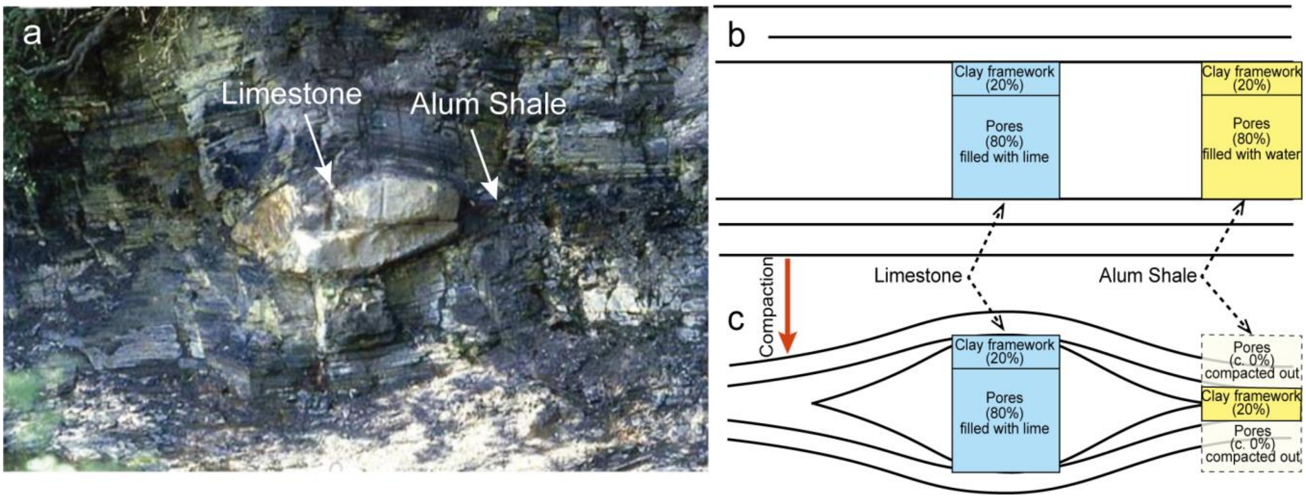

Supplementary Fig. 3 Thickness correction model. **a** Limestone lens in the Alum Shale Formation, Læså, Bornholm. **b** Deposition of Alum Shale, some of which is impregnated with lime cement at an early stage. **c** After compaction, the lime content (~80% in volume) filling the pore spaces prevented the limestone lens from being compacted, while the laterally correlative shale mud was compressed by squeezing out the pore water (~80% in volume). Accordingly, the limestone intervals are reduced to 20% of the thickness prior to analyzing the data.

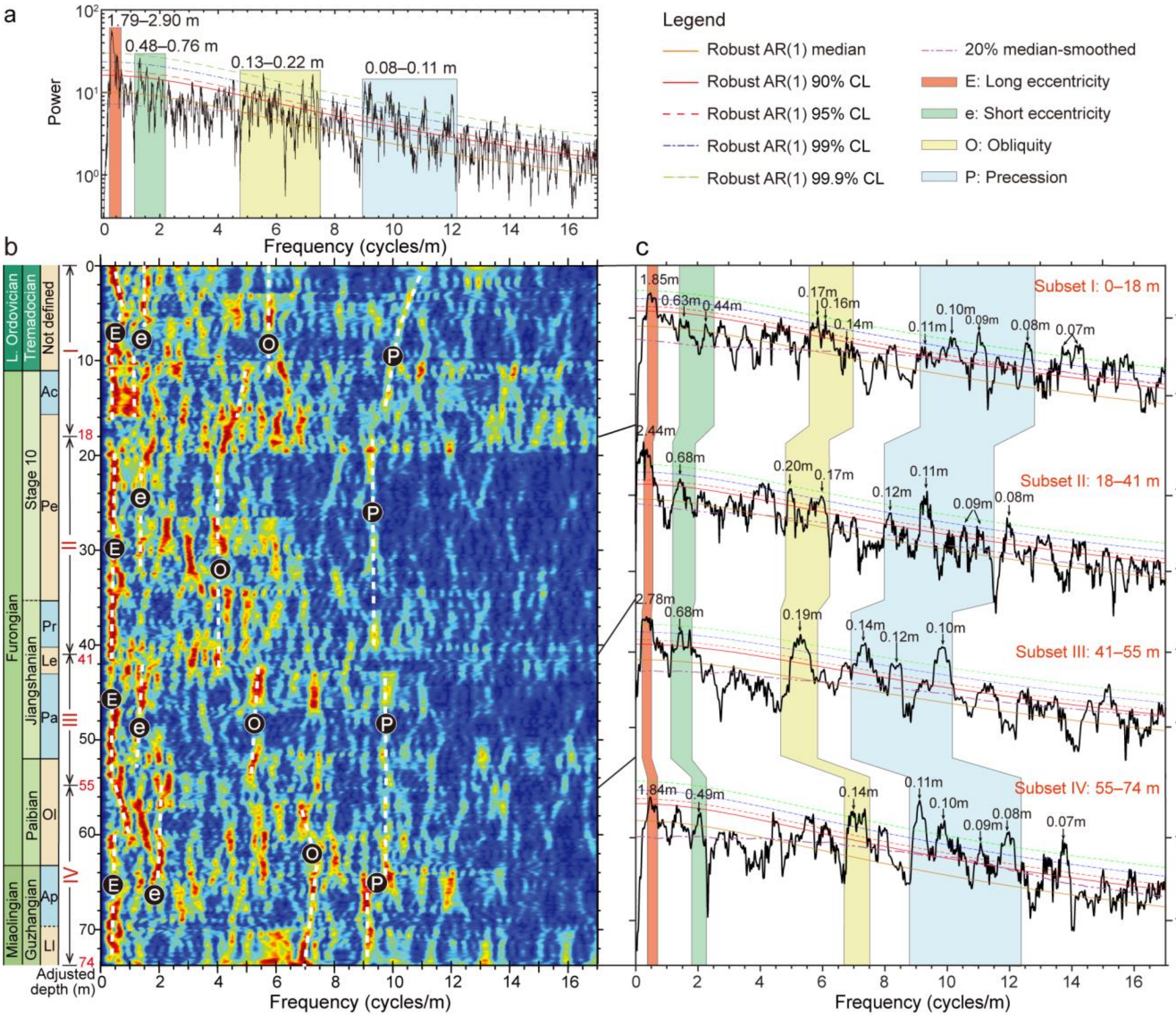

Supplementary Fig. 4 Power spectra of the untuned Al series. **a** 3π MTM power spectrum of the entire untuned Al series after subtracting a 10% "LOESS" trend. **b** The evoFFT spectrum of the Al series with 4 m sliding window and 0.2 m step length. Stage / superzone / zone abbreviations as in Supplementary Fig. 1. Two different horizons are candidates for defining the base of Stage 10 (Supplementary Table. 1); only the base defined by the FAD of *Lotagnostus americanus* is plotted here (see text for details). **c** 3π MTM power spectrum of the untuned Al series for the four subsets 0–18 m, 18–41 m, 41–55 m, 55–74 m after subtracting "LOESS" trends of 35%, 50%, 80% and 35%, respectively. CL= Confidence level. For each subset, long eccentricity, short eccentricity, obliquity and precession were identified and highlighted.

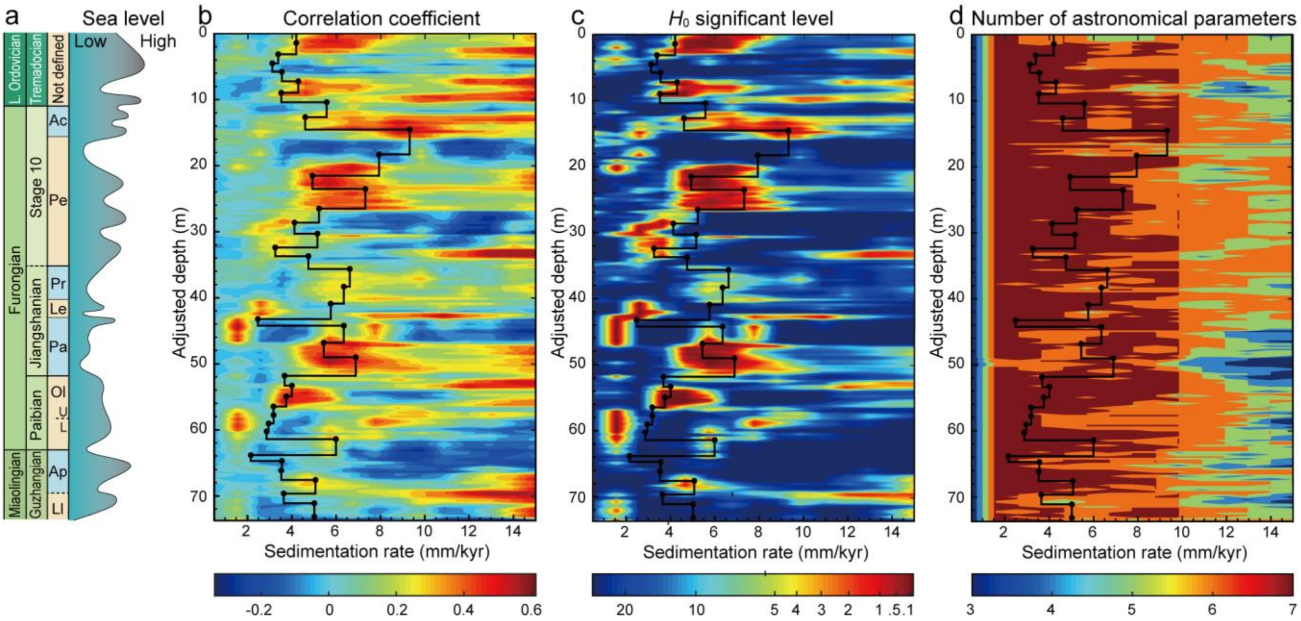

Supplementary Fig. 5 Sea level changes and eCOCO sedimentation rate map based on the Al series. **a** Sea level changes during the Alum Shale deposition[2,49,52]. **b–d** Evolutionary correlation efficient, H_0 significance level and map of the number of contributing astronomical parameters shown with sedimentation rates picked from the minima of 405-kyr cycles (black line, data from Supplementary Table 1). The eCOCO analysis was conducted with a 4 m sliding window, 0.01 m steps and 5000 Monto Carlo simulations. Sedimentation rates range from 0.5 to 15 mm/kyr with a step of 0.1 mm/kyr. The base of Stage 10 defined as in Supplementary Fig. 4. Stage / superzone / zone abbreviations as in Supplementary Fig. 1.

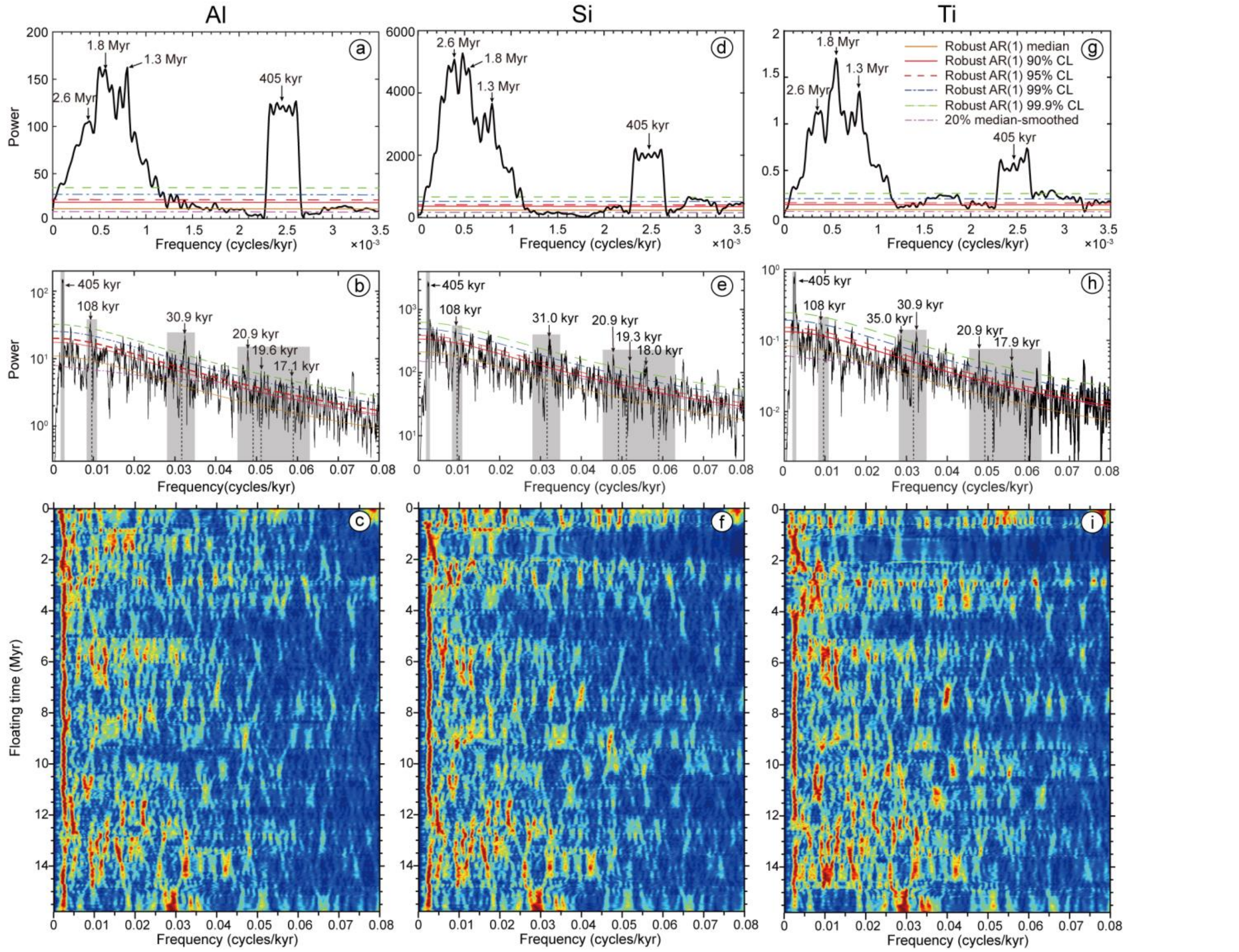

Supplementary Fig. 6 Spectrum analysis of the less diagenesis sensitive Al, Si and Ti series. The Si and Ti series were tuned using the age model established for the Al series. Their consistent correlation corroborates the reliability of our age model. **a–c** 3π MTM spectrum of 405-kyr calibrated Al series after subtracting 8% (a) and 45% (b) "LOESS" trends, respectively, as well as the evoFFT spectrum with 1,000 kyr sliding window and 30 kyr step (c). **d–f** 3π MTM spectrum of 405-kyr calibrated Si series after subtracting 8% (d) and 45% (e) "LOESS" trends, respectively, as well as the evoFFT spectrum with 1,000 kyr sliding window and 30 kyr step (f). **g–i** 3π MTM spectrum of 405-kyr calibrated Ti series after subtracting 8% (g) and 45% (h) "LOESS" trends respectively, as well as the evoFFT spectrum with 1,000 kyr sliding window and 30 kyr step (i). CL=Confidence levels. Grey boxes in panels (b, e and h) indicate the expected astronomical frequencies at 492 Ma, as calculated by Waltham[32].

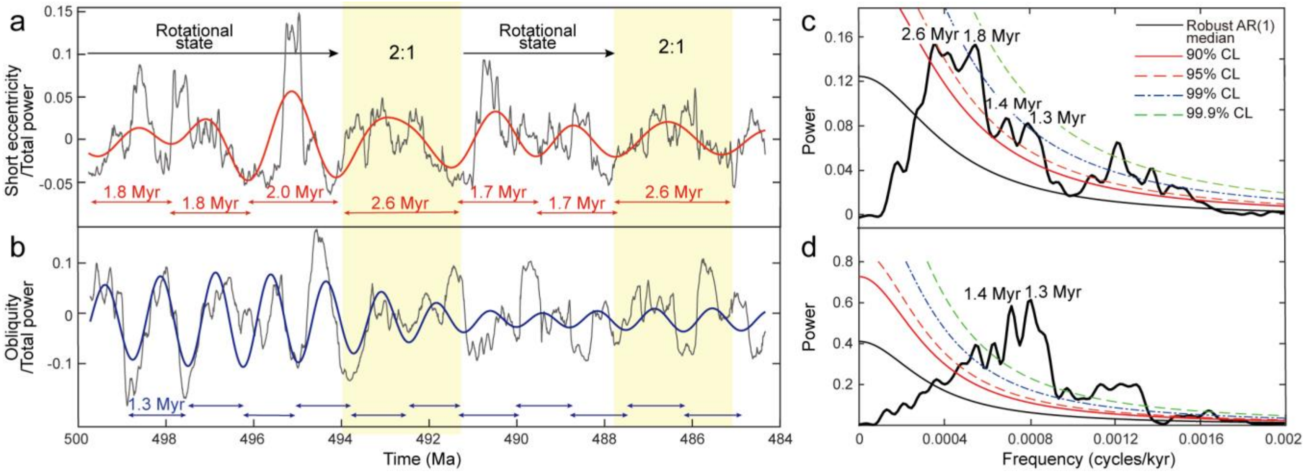

Supplementary Fig. 7 The power ratios of the variance of observed astronomical components to total variance and their power spectra. **a**–**b** The ratios of short eccentricity power/total power and obliquity power/total power were integrated with paired frequency bands of 1/128–1/88 cycles/kyr and 1/45–1/25 cycles/kyr from time-calibrated Al series, respectively, and the total power was integrated from frequency 0 to 0.1 cycles/kyr over a 500 kyr sliding window. **c**–**d** MTM spectra of the power ratios shown in a and b, respectively. CL=Confidence level. Filtered amplitude modulations for the short eccentricity cycles (red line in panel a) and obliquity cycles (blue line in panel b) were extracted using Taner filters with centres (cut-offs) of 0.00047 (0.00030–0.00069) cycles/kyr and 0.00078 (0.00070–0.00090) cycles/kyr, respectively.

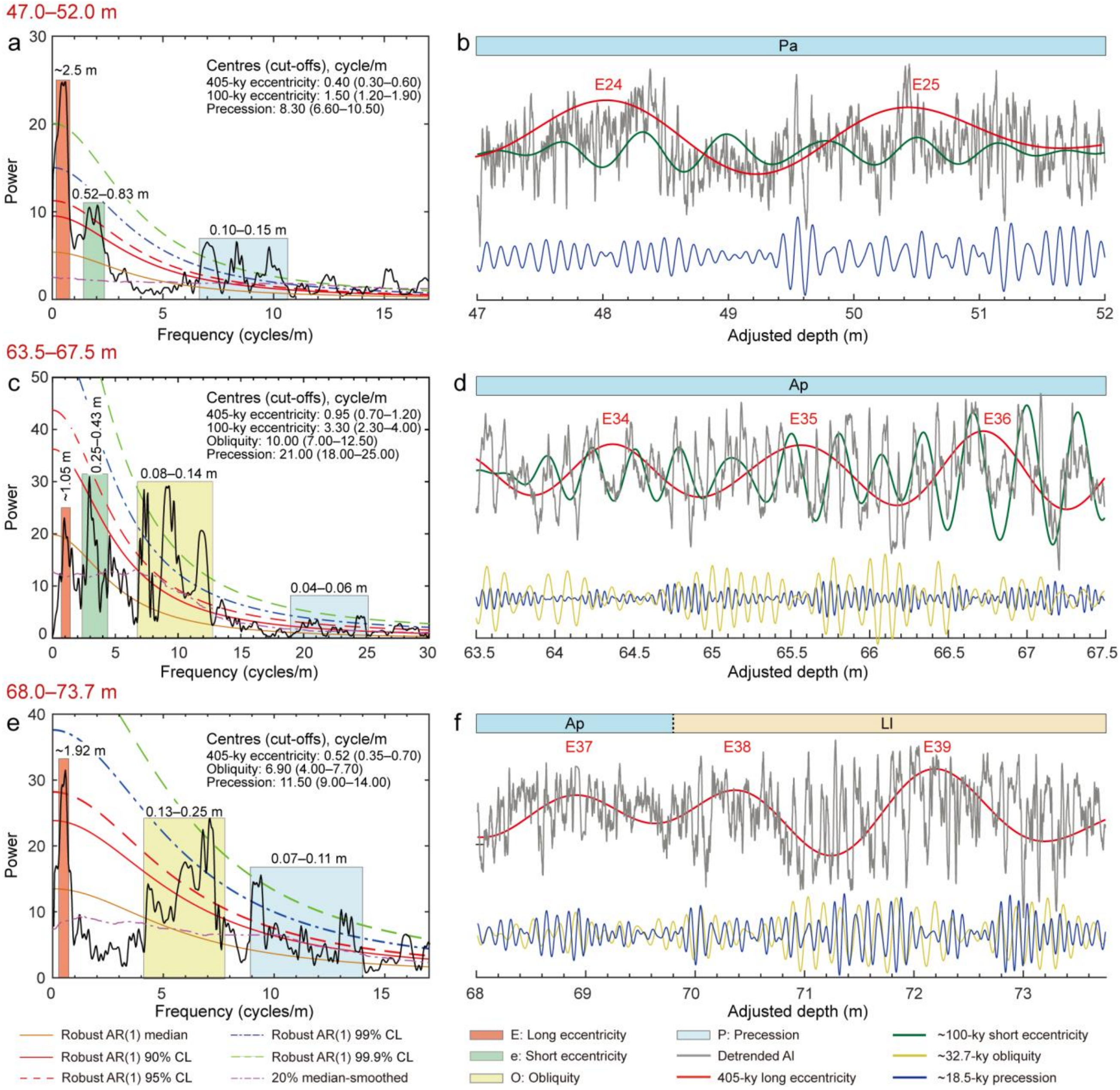

Supplementary Fig. 8 Detailed cyclostratigraphic analysis of three intervals in the *Agnostus pisiformis* Zone and the *Olenus* Superzone. **a, c, e** MTM spectrum of the untuned Al series are shown for the intervals 47.0–52.0 m, 63.5–67.5 m and 68.0–73.7 m. **b, d, f** Filter outputs of long eccentricity, short eccentricity, obliquity and precession that are shown in panels a, c and e, respectively. Superzone / zone abbreviations as in Supplementary Fig. 1.

References

1 Nielsen, A. T., Weidner, T., Terfelt, F. & Hoyberget, M. Upper Cambrian (Furongian) biostratigraphy in Scandinavia revisited: definition of superzones. *GFF* **136**, 193–197 (2014).

2 Nielsen, A. T., Høyberget, M. & Ahlberg, P. The Furongian (upper Cambrian) Alum Shale of Scandinavia: revision of zonation. *Lethaia* **53**, 1–24 (2020).

3 Nielsen, A. T. & Schovsbo, N. H. Cambrian to basal Ordovician lithostratigraphy in southern Scandinavia. *Bull. Geol. Soc. Den.* **53**, 47–92 (2007).

4 Nielsen, A. T., Schovsbo, N. H., Klitten, K., Woollhead, D. & Rasmussen, C. M. Gamma-ray log correlation and stratigraphic architecture of the Cambro–Ordovician Alum Shale Formation on Bornholm, Denmark: Evidence for differential syndepositional isostasy. *Bull. Geol. Soc. Den.* **66**, 237–273 (2018).

5 Westergård, A. H. Stratigraphic results of the borings through the Alum Shales of Scania made in 1941–1942. *Lunds Geologiska Fältklubb*, 185–204 (1942).

6 Westergård, A. H. Borrningar genom Skånes alunskiffer 1941–42. in *Sver. Geol. Unders.* Vol C459 1–45 (Stockholm, 1944).

7 Terfelt, F., Ahlberg, P. E. R. & Eriksson, M. E. Complete record of Furongian polymerid trilobites and agnostoids of Scandinavia - a biostratigraphical scheme. *Lethaia* **44**, 8–14 (2011).

8 Babcock, L. E., Peng, S. C., Geyef, G. & Shergold, J. H. Changing perspectives on Cambrian chronostratigraphy and progress toward subdivision of the Cambrian system. *Geosci. J.* **9**, 101–106 (2005).

9 Peng, S. C. *et al.* Intraspecific variation and taphonomic alteration in the Cambrian (Furongian) agnostoid *Lotagnostus americanus*: new information from China. *Bull. Geosci.*, 281–306 (2015).

10 Landing, E., Westrop, S. R. & Miller, J. F. Globally practical base for the uppermost Cambrian (Stage 10): FAD of the conodont *Eoconodontus notchpeakensis* and the Lawsonian Stage. in *The 15th Field Conference of the Cambrian Stage Subdivision Working Group, International Subcommission on Cambrian Stratigraphy* (eds O. Fatka & P Budil) 18(Czech Geological Survey, Prague, 2010).

11 Landing, E., Westrop, S. R. & Adrain, J. M. The Lawsonian Stage – the *Eoconodontus notchpeakensis* FAD and HERB carbon isotope excursion define a globally correlatable terminal Cambrian stage. *Bull. Geosci.*, 621–640 (2011).

12 Terfelt, F., Eriksson, M. E., Ahlberg, P. & Babcock, L. E. Furongian Series (Cambrian) biostratigraphy of Scandinavia – a revision. *Norwegian Journal of Geology* **88**, 73–87. (2008).

13 Ahlberg, P. & Terfelt, F. Furongian (Cambrian) agnostoids of Scandinavia and their implications for intercontinental correlation. *Geol. Mag.* **149**, 1001–1012 (2012).

14 Nielsen, A. T., Høyberget, M. & Ahlberg, P. The Furongian (upper Cambrian) Alum Shale of Scandinavia: revision of zonation. *Lethaia* **53**, 462–485 (2020).

15 Westergård, A. H. *Sveriges olenidskiffer*. 1–205 (Sveriges Geologiska Undersökning Ca18, Uppsala, 1922).

16 Westergård, A. H. Supplementary notes on the upper Cambrian trilobites of Sweden. *Sveriges Geologiska Undersökning C489*, 1–34 (1947).

17 Ahlberg, P. & Ahlgren, J. Agnostids from the Upper Cambrian of Västergötland, Sweden. *GFF* **118**, 129–140 (1996).

18 Bagnoli, G. & Stouge, S. Upper Furongian (Cambrian) conodonts from the Degerhamn quarry road section, southern Öland, Sweden. *GFF* **136**, 436–458 (2014).

19 Peng, S. C. *et al.* Global Standard Stratotype-Section and Point (GSSP) for the Base of the Jiangshanian Stage (Cambrian: Furongian) at Duibian, Jiangshan, Zhejiang, Southeast China. *Episodes* **35**, 462-477 (2012).

20 Ahlberg, P. *et al.* Integrated Cambrian biostratigraphy and carbon isotope chemostratigraphy of the Grönhögen-2015 drill core, Öland, Sweden. *Geol. Mag.* **156**, 935–949 (2018).

21 Peng, S. C. *et al.* The Global Boundary Stratotype Section and Point (GSSP) of the Guzhangian Stage (Cambrian) in the Wuling Mountains, Northwestern Hunan, China. *Episodes* **32**, 41–55 (2009).

22 Axheimer, N. & Ahlberg, P. A core drilling through Cambrian strata at Almbacken, Scania, S. Sweden: trilobites and stratigraphical assessment. *GFF* **125**, 139–156 (2003).

23 Davidek, K. *et al.* New uppermost Cambrian U–Pb date from Avalonian Wales and age of the Cambrian–Ordovician boundary. *Geol. Mag.* **135**, 303–309 (1998).

24 Schmitz, M. D. Radiometric ages used in GTS2012. in *The Geologic Time Scale 2012* (eds F. M. Gradstein, J. G. Ogg, M. D. Schmitz, & G. M. Ogg) 1045–1082 (Elsevier, Amsterdam, Netherlands, 2012).

25 Schmitz, M. D. Radioisotopic ages used in GTS2020. in *Geologic Time Scale 2020* (eds F. M. Gradstein, J. G. Ogg, M. D. Schmitz, & G. M. Ogg) 1285–1349 (Elsevier, Amsterdam, Netherlands, 2020).

26 Peng, S. C., Babcock, L. E. & Ahlberg, P. The Cambrian Period. in *Geologic Time Scale 2020* (eds Felix M. Gradstein, James G. Ogg, Mark D. Schmitz, & Gabi M. Ogg) 565–629 (Elsevier, Amsterdam, Netherlands, 2020).

27 Landing, E. *et al.* Cambrian–Ordovician boundary age and duration of the lowest Ordovician Tremadoc Series based on U–Pb zircon dates from Avalonian Wales. *Geol. Mag.* **137**, 485–494 (2000).

28 Goldman, D. *et al.* The Ordovician Period. in *Geologic Time Scale 2020* (eds F.M. Gradstein, J.G. Ogg, M.D. Schmitz, & G.M. Ogg) 631–694 (Elsevier, Amsterdam, Netherlands, 2020).

29 Laskar, J. *et al.* A long-term numerical solution for the insolation quantities of the Earth. *Astron. Astrophys.* **428**, 261–285 (2004).

30 Laskar, J. Astrochronology. in *Geologic Time Scale 2020* (eds F.M. Gradstein, J.G. Ogg, M.D. Schmitz, & G.M. Ogg) 139–158 (Elsevier, Amsterdam, Netherlands, 2020).

31 Berger, A., Loutre, M. F. & Laskar, J. Stability of the Astronomical Frequencies over the Earths History for Paleoclimate Studies. *Science* **255**, 560–566 (1992).

32 Waltham, D. Milankovitch period uncertainties and their impact on cyclostratigraphy. *J. Sediment. Res.* **85**, 990–998 (2015).

33 Berger, A. & Loutre, M. F. Astronomical forcing through geological time. in *Orbital Forcing and Cyclic Sequences: International Association of Sedimentologists* (eds P.L. Boer & D.G. Smith) 15–24 (Blackwell Scientific Publications, Oxford, 1994).

34 Hinnov, L. A., Wu, H. C. & Fang, Q. Reply to the comment on "Geologic evidence for chaotic behavior of the planets and its constraints on the third-order eustatic sequences at the end of the Late Paleozoic Ice Age" by Fang, Q, Wu, H., Hinnov, L. A., Jing, X., Wang, X., & Jiang, Q [Palaeogeography, Palaeoclimatology, Palaeoecology, Volume 440, 15 December 2015, 848–859 (2015)]. *Palaeogeogr. Palaeoclimatol. Palaeoecol.* **461**, 475–480 (2016).

35 Hinnov, L. A. Cyclostratigraphy and Astrochronology in 2018. in *Stratigraphy and Time Scales* Vol 3 (ed M. Montenari) 1–80 (Academic Press, Amsterdam, 2018).

36 Schovsbo, N. H. The geochemistry of Lower Palaeozoic sediments deposited on the margins of Baltica. *Bull. Geol. Soc. Den.* **50**, 11–27 (2003).

37 Li, M. S., Kump, L. R., Hinnov, L. A. & Mann, M. E. Tracking variable sedimentation rates and astronomical forcing in Phanerozoic paleoclimate proxy series with evolutionary correlation coefficients and hypothesis testing. *Earth Planet. Sci. Lett.* **501**, 165–179 (2018).

38 Nielsen, A. T. & Schovsbo, N. H. The regressive Early-Mid Cambrian 'Hawke Bay Event' in Baltoscandia: Epeirogenic uplift in concert with eustasy. *Earth Sci. Rev.* **151**, 288–350 (2015).

39 Hinnov, L. A. New perspectives on orbitally forced stratigraphy. *Annu. Rev. Earth Planet. Sci.* **28**, 419–475 (2000).

40 Laskar, J. The Chaotic Motion of the Solar-System - a Numerical Estimate of the Size of the Chaotic Zones. *Icarus* **88**, 266–291 (1990).

41 Laskar, J., Fienga, A., Gastineau, M. & Manche, H. La2010: a new orbital solution for the long-term motion of the Earth. *Astron. Astrophys.* **532** (2011).

42 Hinnov, L. A. Cyclostratigraphy and its revolutionizing applications in the earth and planetary sciences. *Geol. Soc. Am. Bull.* **125**, 1703–1734 (2013).

43 Wu, H. C. *et al.* An similar to 34 m.y. astronomical time scale for the uppermost Mississippian through Pennsylvanian of the Carboniferous System of the Paleo–Tethyan realm. *Geology* **47**, 83–86 (2019).

44 Crampton, J. S. *et al.* Pacing of Paleozoic macroevolutionary rates by Milankovitch grand cycles. *Proc. Natl. Acad. Sci. U. S. A.* **115**, 5686–5691 (2018).

45 Zhong, Y. Y. *et al.* Late Ordovician obliquity-forced glacio-eustasy recorded in the Yangtze Block, South China. *Palaeogeogr. Palaeoclimatol. Palaeoecol.* **540**, 1–16 (2020).

46 Fang, Q. *et al.* Geologic evidence for chaotic behavior of the planets and its constraints on the third-order eustatic sequences at the end of the Late Paleozoic Ice Age. *Palaeogeogr. Palaeoclimatol. Palaeoecol.* **440**, 848–859 (2015).

47 Da Silva, A. C. *et al.* Refining the Early Devonian time scale using Milankovitch cyclicity in Lochkovian–Pragian sediments (Prague Synform, Czech Republic). *Earth Planet. Sci. Lett.* **455**, 125–139 (2016).

48 Fang, Q. *et al.* Abiotic and biotic responses to Milankovitch-forced megamonsoon and glacial cycles recorded in South China at the end of the Late Paleozoic Ice Age. *Global and Planetary Change* **163**, 97–108 (2018).

49 Sørensen, A. L. *et al.* Astronomically forced climate change in the late Cambrian. *Earth Planet. Sci. Lett.* **548**, 1–13 (2020).

50 Nielsen, A. T. & Andersen, L. F. Furongian (upper Cambrian) trilobites and agnostoids from the Alum Shale Formation of Bornholm, Denmark: revised taxonomy and biostratigraphy. *Bull. Geol. Soc. Den.* **69**, 123–213 (2021).

51 Zhao, Z. *et al.* High-resolution carbon isotope chemostratigraphy of the middle Cambrian to lowermost Ordovician in southern Scandinavia: Implications for global correlation. *Global and Planetary Change* (2022).

52 Rasmussen, C. M. Ø., Kröger, B., Nielsen, M. L. & Colmenar, J. Cascading trend of Early Paleozoic marine radiations paused by Late Ordovician extinctions. *Proc. Natl. Acad. Sci. U. S. A.* **116**, 7207–7213 (2019).
