# Supplementary material for: Synchronizing rock clocks in the late Cambrian
Source: Nat Commun. 2022 Apr 13;13:1990. doi: 10.1038/s41467-022-29651-4 (PMC9007955; doi:10.1038/s41467-022-29651-4)
Supplement: Supplementary file 2 — Source Data [file 41467_2022_29651_MOESM2_ESM.zip › Read me.docx]

**Read me file for data storage linked to paper**

*"Synchronizing rock clocks in the late Cambrian" by Zhengfu Zhao, Nicolas Thibault, Tais W. Dahl, Niels H. Schovsbo, Aske L. Sørensen, Christian M.Ø. Rasmussen, and Arne T. Nielsen*

The following data are stored in the folder:

1. All Core scanning XRF concentration data and supplementary handheld-XRF concentration data for the broken intervals (accounting for only ~2.5 m of the 77-m-long core), limestone is included. The HH-XRF concentration in broken intervals have been calibrated to XRF-CS concentration as described in the Methods section.

*S1a. XRF-CS data_Albjära-1 core*

*S1b. HH-XRF data_Albjära-1 core*

2. Corrected HH-XRF concentrations are incorporated to XRF-CS archive to get a merged XRF concentration dataset. Adjusted depths were obtained by measuring from the Alum Shale Formation top downward after reducing the limestone thickness to 20% (as described in Methods section), then interpolating with 1 mm sampling resolution.

*S2. Merged XRF data­_Albjära-1 core (used in this study)*

3. Gamma-ray log data

*S3. Gamma-ray log_Albjära-1 core*

4. Carbon isotope data

*S4. Carbon isotopes_Albjära-1 & Gislövshammar-2 cores*
